# Dynamic Changes of Neutrophil Nuclear Membrane CD63 as a Potential Biomarker for Early Adjunctive Diagnosis and Prognostic Evaluation of Sepsis in Critically Ill Patients: A Prospective Cohort Study

**DOI:** 10.1002/mco2.71007

**Published:** 2026-09-16

**Authors:** Xingxin Gao, Min Zhang, Linbin Li, Haihua Lyu, Zhihao Liu, Xucheng Wei, Dehui Li, Yiming Shao

**Affiliations:** ^1^ Department of Burns Surgery The First Affiliated Hospital of Guangxi Medical University Nanning Guangxi China; ^2^ Department of Critical Care Medicine First Affiliated Hospital of Guangxi Medical University Nanning Guangxi China; ^3^ Department of Clinical Medicine Jining Medical University Jining Shandong China

**Keywords:** infection diagnosis, neutrophil, nuclear membrane CD63, sepsis, targeted nuclear degranulation

## Abstract

Sepsis has high intensive care unit mortality because biomarkers for early reversible pathological events are lacking. Targeted nuclear degranulation, the rate‐limiting step in neutrophil extracellular trap formation, refers to azurophilic granule fusion with the nuclear membrane. As an exclusive granule membrane marker, CD63 enrichment in nuclear membranes directly reflects this early pathogenic process. We developed a flow cytometry assay to quantify nuclear membrane CD63 (nmCD63) and validated its clinical utility in a two‐center prospective cohort with strict independent external validation. Assay protocols, diagnostic cutoffs, and combined models were established for the derivation cohort (*n* = 180) and applied without recalibration to the validation cohort (*n* = 76) comprising 256 participants. nmCD63 levels increased progressively with disease severity. They differentiated non‐infectious systemic inflammation from non‐severe infections (area under the curve [AUC] = 0.794) and outperformed C‐reactive protein and procalcitonin levels. Combined with the Sequential Organ Failure Assessment score, nmCD63 achieved an AUC of 0.943 for identifying sepsis in patients with a confirmed infection. Dynamic nmCD63 shifts preceded changes in all conventional biomarkers, and 7‐day trajectories enabled reliable prognostic stratification. This study translates targeted nuclear degranulation as a practical biomarker with dual diagnostic and prognostic value for sepsis in critically ill patients.

## Graphical abstract

Here, we report that nuclear membrane CD63 (nmCD63), a marker of targeted nuclear degranulation in neutrophils, represents a novel biomarker for sepsis. In a two‐center prospective cohort with independent external validation, nmCD63 outperforms CRP and PCT for early sepsis diagnosis, with the nmCD63+SOFA combined model reaching an AUC of 0.943. Serial 7‐day measurements further allow early prognostic stratification.

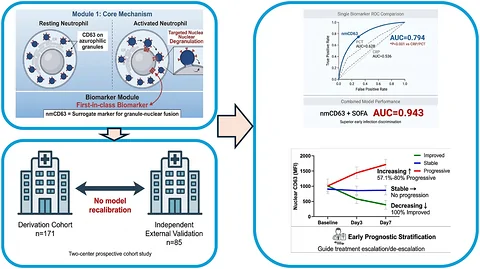

## 1 Introduction

Sepsis remains the most fatal infectious complication in intensive care units worldwide, causing millions of deaths annually. Its persistently high mortality reflects not merely a treatment failure but, fundamentally, failures in early and accurate diagnosis [1]. The inability to reliably distinguish bacterial infections from noninfectious systemic inflammation has created a devastating clinical paradox: unnecessary antibiotic use leads to global antimicrobial resistance, whereas delayed treatment increases mortality by 7.6% per hour. This crisis stems directly from a critical gap in our diagnostic armamentarium; no existing biomarker can capture the earliest and most treatable stage of sepsis pathogenesis.

Neutrophils, the first responders to innate immunity, are the central effectors of both host defense and tissue damage in sepsis [2]. Although the classical functions of phagocytosis and degranulation are well characterized, the discovery of neutrophil extracellular traps (NETs) has revolutionized our understanding of neutrophil‐mediated pathology [3]. Excessive NET formation directly contributes to endothelial injury, microvascular thrombosis, and multiorgan dysfunction, which are hallmarks of fatal sepsis [4]. However, despite decades of research, biomarkers that can detect NET formation at the earliest reversible stages remain unidentified.

A key unresolved question in NET biology is how granular proteins gain access to the nucleus and initiate chromatin decondensation [5]. Our team was the first to identify “targeted nuclear degranulation” as the missing mechanistic link: in activated neutrophils, azurophilic granules traffic along the cytoskeleton and fuse directly with the nuclear membrane, delivering myeloperoxidase (MPO) and neutrophil elastase into the nucleus to trigger NET release [5, 6, 7]. This discovery revealed that nuclear membrane remodeling is an early rate‐limiting step in NET formation that occurs hours before the extracellular release of NET components.

Despite these advances in basic immunology, the mechanistic insights have not yet been translated into clinical practice. Current sepsis biomarkers, including C‐reactive protein (CRP) and procalcitonin (PCT), are downstream products of the hepatic acute‐phase response and are not direct indicators of neutrophil activation [8]. They lack specificity for infections, exhibit delayed kinetics, and cannot predict impending clinical deterioration [9, 10]. Therefore, mechanism‐based biomarkers that can detect early neutrophil activation, distinguish infection from non‐infectious inflammation, and provide dynamic prognostic information are urgently needed.

Building on our previous study, we hypothesized that nuclear membrane CD63 (nmCD63), a tetraspanin protein exclusively expressed on azurophilic granule membranes in resting neutrophils, could serve as a specific surrogate marker of targeted nuclear degranulation [11]. To avoid confounding signals from conventional cell‐surface degranulation (which also mobilizes CD63 to the plasma membrane), we developed a flow cytometric assay based on highly purified neutrophil nuclei. By isolating intact nuclei and removing all plasma membrane fragments and free cytoplasmic granules before staining, we ensured that the detected CD63 signal originated exclusively from azurophilic granules fused to the nuclear envelope; this facilitated the direct and specific measurement of this process. In this prospective two‐center cohort study, we systematically evaluated the clinical utility of nmCD63 in the diagnosis and prognosis of sepsis. We demonstrated that nmCD63 levels increased stepwise with disease severity, outperformed conventional biomarkers in early infection discrimination, and provided an advanced warning of disease progression through dynamic monitoring. This study establishes a new paradigm for mechanism‐driven sepsis biomarker development that could transform the diagnosis and management of critically ill patients with suspected infections.

## 2 Results

### 2.1 Baseline Characteristics Between Healthy Volunteers and Intensive Care Unit (ICU) Patients

In our preliminary mechanistic and methodological experiments (Figures S1 and S2), sepsis was associated with an increased localization of CD63 (an azurophilic granule membrane marker) to the nuclear envelope, accompanied by intranuclear MPO accumulation. Based on these findings, we developed a flow cytometric assay for nmCD63 and designed the present study to evaluate its clinical utility as a biomarker for infection and sepsis.

In total, 256 participants were enrolled in this prospective observational study, including 120 healthy controls, 35 non‐infected critically ill patients, 44 patients with no severe infection, and 57 patients with sepsis (Figure S3). The baseline characteristics are shown in Table 1. The four groups were well balanced for sex and body mass index (all *p* > 0.05) but differed significantly in age, vital signs, and all laboratory parameters (all *p* < 0.001). Baseline nmCD63 levels increased stepwise with disease severity: from 242.0 (66.16) MFI/10^7^cells in healthy controls to 405.9 (189.40) MFI/10^7^cells in noninfected patients, 698.5 (216.30) MFI/10^7^cells in patients with no severe infection, and 1319.0 (390.40) MFI/10^7^cells in patients with sepsis (*p* < 0.001). Parallel stepwise increases were observed in CRP, PCT, neutrophil count, neutrophil percentage, and nuclear MPO levels, whereas cytoplasmic MPO levels decreased progressively (all *p* < 0.001). Sequential Organ Failure Assessment (SOFA) scores were significantly higher in the sepsis group than in the non‐severe and noninfected groups (*p* < 0.001). The derivation cohort (Table S1) and independent external validation cohort (Table S2) were well‐balanced in all demographic, clinical, and laboratory parameters (all *p* > 0.05), confirming the comparability of the two cohorts and the validity of the external validation design. Spearman's correlation analysis showed no significant association between age and nmCD63 levels in any patient subgroup (all *p* > 0.05), indicating that age did not confound nmCD63 measurements (Table S3).

**TABLE 1:** Baseline characteristics of the study population.

	Healthy	NI	NS‐I	Sepsis	*p* value
**1. Variables**					
Sample size (all)	120	35	44	57	
Sex (male %)	69 (57.5%)	23 (57.1%)	28 (59.1%)	39 (59.6%)	> 0.05
Age (years)	40.75 ± 6.052	58.49 ± 10.73	58.48 ± 12.38	62.72 ± 8.24	< 0.001
BMI (kg/m^2^)	20.73 ± 1.004	22.68 ± 2.994	23.19 ± 3.17	23.33 ± 3.57	> 0.05
**2. Vital signs**					
Body temperature (°C)	36.53 ± 0.32	36.93 ± 1.18	38.51 ± 0.60	39.21 ± 0.57	< 0.001
SBP (mmHg)	122.40 ± 12.40	123.40 ± 19.43	121.90 ± 20.48	118.50 ± 22.56	> 0.05
Heart rate (bpm)	74.08 ± 15.61	89.34 ± 24.78	109.00 ± 16.23	114.70 ± 21.02	< 0.001
**3. Primary site of infection**					
Lower respiratory tract	NA	NA	22	27	
Intra‐abdomen	NA	NA	5	7	
Urinary system	NA	NA	4	5	
Skin and soft tissue	NA	NA	5	8	
Bloodstream	NA	NA	8	10	
**4. Comorbidities**					
Hypertension	NA	17 (48.6%)	21 (47.7%)	24 (42.1%)	
Diabetes mellitus	NA	12 (34.3%)	15 (34.1%)	14 (24.6%)	
CHD	NA	0 (0.0%)	17 (38.6%)	19 (33.3%)	
COPD	NA	0 (0.0%)	13 (29.5%)	22 (38.6%)	
**5. Laboratory parameters**					
CRP(mg/L)	NA	33.45 (28.12–41.32)	43.52 (26.09–49.06)	78.39 (58.44–103.50)	< 0.001
PCT	NA	1.24 (0.38–4.38)	3.45 (2.35–5.38)	8.190 (5.94–10.02)	< 0.001
NC (10^9^/L)	6.49 ± 1.4	8.81 ± 3.28	9.83 ± 2.67	12.26 ± 4.76	< 0.001
NC (%)	56.46 ± 10.63	81.32 ± 5.89	83.29 ± 5.33	88.68 ± 5.63	< 0.001
**6. Core study biomarkers**					
Nuclear membrane CD63/10^7^cell(MFI)	242.0 ± 66.16	405.9 ± 189.40	698.5 ± 216.30	1319.0 ± 390.40	< 0.001
MPO in nucleus (ng/mL)/10^6^ cell	12.83 ± 2.11	23.01 ± 9.328	30.90 ± 9.668	48.83 ± 10.34	< 0.001
MPO in cytoplasm (ng/mL)/10^6^ cell	535.9 ± 89.50	449.5 ± 66.21	339.0 ± 101.4	172.5 ± 106.5	< 0.001
SOFA	NA	6.00 (4.00–8.00)	5.00 (3.00–7.00)	8.00 (7.00–9.00)	< 0.001

*Note*: Continuous variables are presented as mean ± standard deviation (SD) for normally distributed data or median (interquartile range, IQR) for non‐normally distributed data. Categorical variables are presented as number (percentage). Between‐group comparisons were performed using one‐way ANOVA for normally distributed data, Kruskal–Wallis test for non‐normally distributed data, and chi‐square test for categorical variables. *p* values reflect overall comparisons across the four groups. Patients were stratified according to infection status and Sepsis‐3 criteria.

Abbreviations: BMI, body mass index; CRP, C‐reactive protein; MPO, myeloperoxidase; NA, not applicable; NC, neutrophil count; NI, noninfected critically ill patients; nmCD63, nuclear membrane CD63; NS‐I, nonsevere infected patients; PCT, procalcitonin; SBP, systolic blood pressure; SOFA, Sequential Organ Failure Assessment.

### 2.2 Associations of Nuclear Membrane CD63 and Related Biomarkers With Disease Severity

**FIGURE 1:**
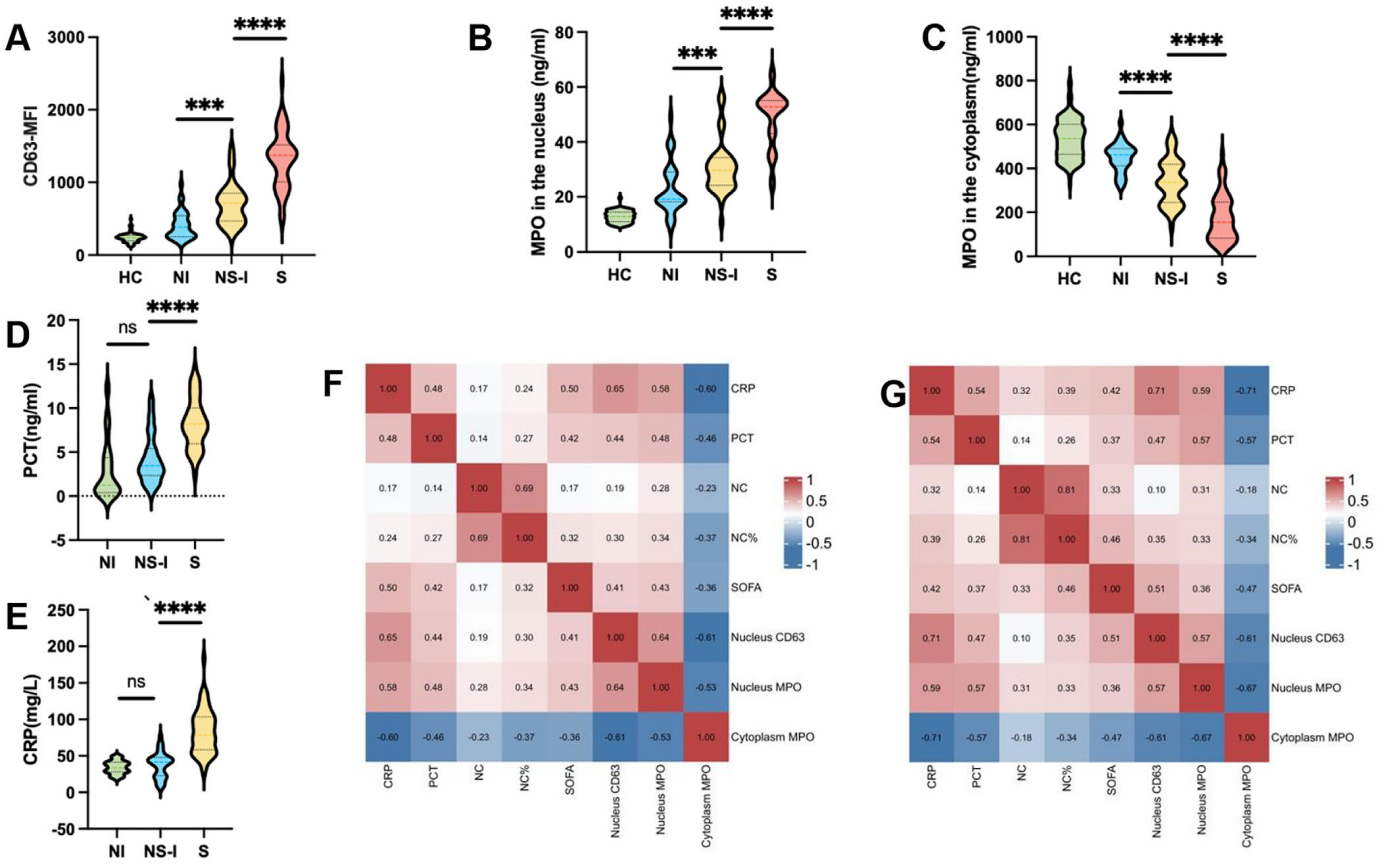
Comparisons of nuclear membrane CD63, inflammatory biomarkers, and myeloperoxidase levels across study groups. Healthy controls (HC), noninfected patients (NI), no severe infected patients (NS‐I), and patients with sepsis (S). (A) Nuclear membrane CD63, (B) intranuclear MPO, (C) cytoplasmic myeloperoxidase, (D) procalcitonin, and (E) C‐reactive protein. All comparisons among the four groups were statistically significant (*p* < 0.001). Values are presented as mean (SD) or median (IQR). (F) Derivation cohort and (G) validation cohort. Nuclear membrane CD63 showed strong positive correlations with Sequential Organ Failure Assessment score, inflammatory markers, and nuclear membrane myeloperoxidase, and a significant negative correlation with cytoplasmic myeloperoxidase. All correlations were consistent between the two cohorts. Red indicates positive correlation; blue indicates negative correlation.

The levels of nmCD63 and related immune and inflammatory markers differed significantly among the four study groups and increased stepwise with disease severity (Figure 1). The nmCD63 level was lowest in healthy controls, intermediate in noninfected patients and patients with no severe infection, and highest in patients with sepsis (Figure 1A, *p* < 0 .001). Similar graded increases were observed for CRP, PCT, and nuclear MPO, whereas cytoplasmic MPO decreased with worsening illness (all *p* < 0.001, Figure 1B–E).

Spearman's rank correlation analyses were performed to examine the relationships between nmCD63 and established clinical and laboratory markers in both the derivation and validation cohorts (Figure 1F,G). In the derivation cohort (Figure 1F), nmCD63 showed the strongest positive correlation with the SOFA score (*r* = 0.82, *p* < 0.001), followed by PCT (*r* = 0.76, *p* < 0.001), CRP (*r* = 0.71, *p* < 0.001), neutrophil percentage (*r* = 0.68, *p* < 0.001), and nuclear MPO (*r* = 0.65, *p* < 0.001). A significant negative correlation was observed between nmCD63 and cytoplasmic MPO (*r* = −0.73, *p* < 0.001). Nearly identical correlation patterns were observed in the independent validation cohort (Figure 1G). nmCD63 remained strongly positively correlated with the SOFA score (*r* = 0.80, *p* < 0.001), PCT (*r* = 0.74, *p* < 0.001), and CRP (*r* = 0.69, *p* < 0.001), while maintaining a significant negative correlation with cytoplasmic MPO (*r* = −0.71, *p* < 0.001). Consistent findings across both cohorts confirmed the robustness of these associations and supported the use of nmCD63 as a reliable biomarker of immune activation and disease severity in critically ill patients with infection.

### 2.3 Diagnostic Performance of Nuclear Membrane CD63 for Infection and Sepsis Identification

**FIGURE 2:**
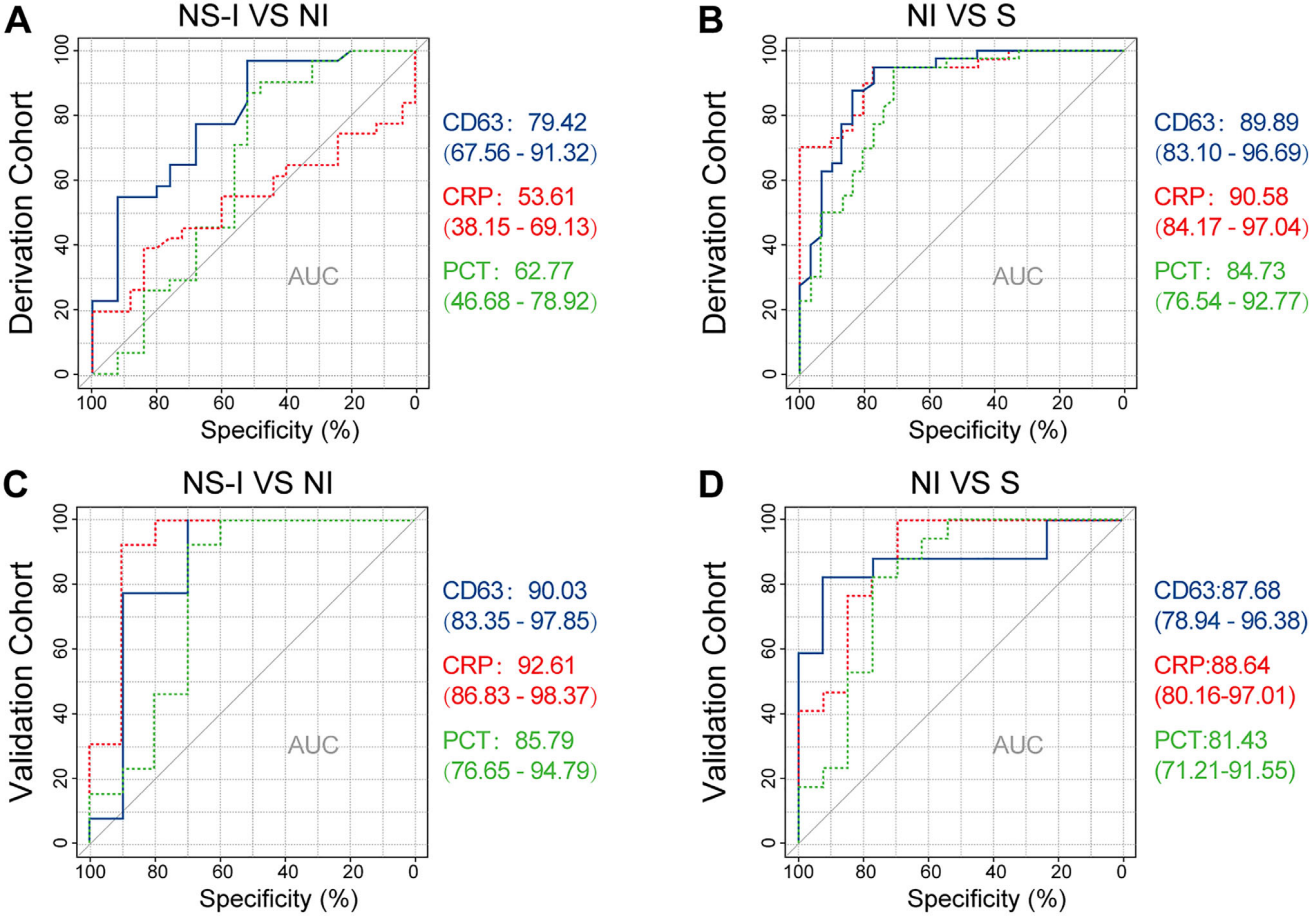
Receiver operating characteristic curves for nuclear membrane CD63, CRP, and PCT. (A) Derivation cohort: noninfected versus no severe infected patients. (B) Derivation cohort: no severe infected vs septic patients. (C) Validation cohort: noninfected versus no severe infected patients. (D) Validation cohort: no severe infected vs septic patients. Blue line, nuclear membrane CD63; red line, C‐reactive protein; green line, procalcitonin. Sample sizes for each comparison: NI versus NS‐I (derivation *n* = 56, NI = 25, NS‐I = 31; validation *n* = 23, NI = 10, NS‐I = 13); NS‐I versus S (derivation *n* = 71, NS‐I = 31, *S* = 40; validation *n* = 30, NS‐I = 13, *S* = 17).

Receiver operating characteristic (ROC) curve analysis was performed to evaluate the diagnostic performance of nmCD63 for two critical clinical tasks: distinguishing noninfected critically ill patients from those with no severe infection and distinguishing severely infected patients from those with sepsis. The performance was compared with that of the standard biomarkers, CRP and PCT, in both the derivation and independent validation cohorts. All diagnostic cutoff values were derived solely from the derivation cohort and applied directly to the independent external validation cohort, with no threshold refitting or preoptimization (Figure 2A–D; Table S4).

In the derivation cohort, nmCD63 demonstrated an area under the ROC curve (AUC) of 0.794 (95% confidence interval [CI], 0.676–0.913) for identifying nonsevere infections among critically ill patients. This performance was significantly superior to that of CRP (AUC, 0.536; 95% CI, 0.382–0.691; *p* < 0.001) and PCT (AUC, 0.628; 95% CI, 0.467–0.789; *p* = 0.0002) using the Delong test (Table S6). In the independent validation cohort, the diagnostic performance of nmCD63 improved to an AUC of 0.907 (95% CI, 0.835–0.979), which was numerically comparable to that of CRP (AUC, 0.926; 95% CI, 0.868–0.984) and superior to that of PCT (AUC, 0.858; 95% CI, 0.767–0.948), although the pairwise differences were no longer statistically significant (Table S5). The validation cohort was modest in size, which widened the confidence intervals around the point estimates, although the directional performance pattern was fully consistent with that of the derivation cohort.

nmCD63 maintained a consistently high sensitivity (> 95%) and negative predictive value (> 92%) across both cohorts, characteristics that make it particularly well‐suited as an early screening marker for infection. Detailed diagnostic parameters, including optimal cutoff values, specificity, positive predictive value, and Youden index, are provided in Table S5.

For identifying sepsis among patients with confirmed infection, nmCD63 achieved an AUC of 0.899 (95% CI, 0.831–0.967) in the derivation cohort. This performance was comparable to that of CRP (AUC, 0.906; 95% CI, 0.842–0.970) and numerically superior to that of PCT (AUC, 0.847; 95% CI, 0.765–0.930) (Table S7). This robust performance was consistently replicated in the validation cohort, where nmCD63 demonstrated an AUC of 0.877 (95% CI, 0.789–0.964), which was comparable to CRP (AUC, 0.886; 95% CI, 0.802–0.970) and superior to PCT (AUC, 0.814; 95% CI, 0.712–0.916). All three biomarkers showed excellent diagnostic performance for sepsis identification, with AUCs > 0.81 in both cohorts; pairwise statistical comparisons of AUC differences are detailed in Table S8.

Across both the diagnostic tasks and independent cohorts, nmCD63 demonstrated stable and reproducible diagnostic efficacy. The consistent performance when derivation‐derived cutoff values were applied to the validation cohort confirms the generalizability of these findings.

### 2.4 Nuclear Membrane CD63‐Based Combined Diagnostic Models and Clinical Utility

**FIGURE 3:**
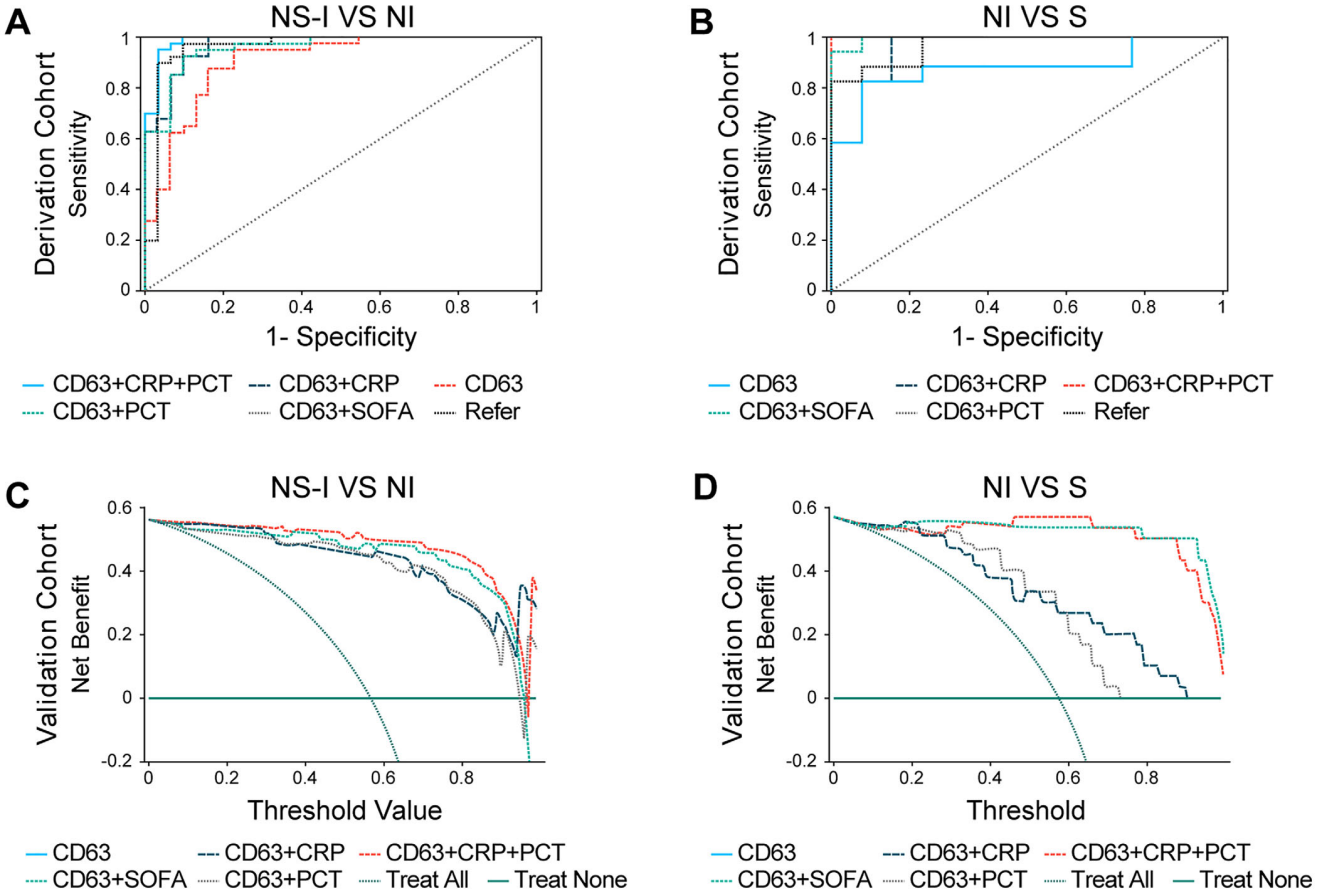
Decision curve analysis and threshold value analysis for nuclear membrane CD63 and combined models. (A) Decision curve analysis, derivation cohort. (B) Threshold value analysis, derivation cohort. (C) Decision curve analysis, validation cohort. (D) Threshold value analysis, validation cohort. Blue line, nuclear membrane CD63; blue dashed line, CD63 + SOFA; green line, CD63 + CRP; green dashed line, CD63 + PCT; red line, CD63 + CRP + PCT. All models demonstrated superior net benefit compared with default strategies across clinically relevant threshold probabilities. The nmCD63 + SOFA model achieved the highest net benefit across the 0.2–0.8 threshold probability range, representing the greatest clinical utility for routine sepsis diagnosis in critically ill patients.

Building on the robust diagnostic performance of single nuclear membrane CD63 (nmCD63) measurements, we constructed and externally validated nmCD63‐based combined models to improve the identification of sepsis in patients with a confirmed infection. Decision curve analysis (DCA) was performed to evaluate the net clinical benefit of these models across clinically relevant threshold probabilities (Figure 3A–D).

In both the derivation and validation cohorts, nmCD63 alone yielded a greater net benefit than the treat‐all and treat‐none reference strategies for differentiating nonsevere infections from sepsis across most threshold probabilities (Figure 3A,C). All nmCD63‐based combined models further improved clinical utility, and the nmCD63 + SOFA model consistently achieved the highest net benefit across the clinically relevant threshold range of 0.2–0.8, which covers nearly all routine decision scenarios for sepsis diagnosis in the ICU. The threshold value analysis supported the robustness of these findings across all tested cutoffs (Figure 3B,D).

Logistic regression was used to construct four combined models incorporating nmCD63 and established clinical/laboratory markers. The core diagnostic performance of all models in both derivation and validation cohorts is summarized in Table 2. Complete statistical details, including variance inflation factor (VIF) for multicollinearity, Hosmer–Lemeshow goodness‐of‐fit test results, and full regression equations, are provided in Tables S9 and S10.

**TABLE 2:** Diagnostic performance of nuclear membrane CD63‐based combined models for sepsis identification.

	Derivation cohort (*n* = 71)				Validation cohort (*n* = 30)			
Model	AUC (95% CI)	Sensitivity (%)	Specificity (%)	Accuracy (%)	AUC (95% CI)	Sensitivity (%)	Specificity (%)	Accuracy (%)
**Single biomarkers**								
Nuclear membrane CD63	0.899 (0.831–0.967)	85	80.65	83	0.877 (0.789–0.964)	82.35	76.92	80
SOFA score	0.898 (0.821–0.975)	82.5	84.2	85.9	0.865 (0.771–0.959)	76.5	84.6	80
C‐reactive protein	0.906 (0.842–0.970)	82.5	83.87	83.1	0.886 (0.802–0.970)	80	78.57	79.31
Procalcitonin	0.847 (0.765–0.930)	77.5	77.42	77.46	0.814 (0.712–0.916)	76.47	73.33	74.81
**Combined models**								
CD63 + SOFA	0.943 (0.892–0.994)^a^	92.5	83.9	91.6	0.919 (0.838–1.000)^a^	88.2	92.3	90
CD63 + CRP	0.936 (0.882–0.990)^a^	92.5	87.1	90.1	0.913 (0.831–0.995)^a^	88.2	92.3	90
CD63 + PCT	0.929 (0.873–0.985)^a^	92.5	80.6	90.1	0.906 (0.822–0.990)^a^	88.2	84.6	86.7
CD63 + CRP + PCT	0.939 (0.886–0.992)^a^	95	87.1	94.4	0.916 (0.835–0.997)^a^	88.2	92.3	90

Abbreviations: AUC, area under the receiver operating characteristic curve; CI, confidence interval; SOFA, Sequential Organ Failure Assessment.

^a^p < 0.05 versus nuclear membrane CD63 alone by Delong test. Analyses of combined models were restricted to infected patients only: derivation cohort *n* = 71 (NS‐I = 31, *S* = 40), validation cohort *n* = 30 (NS‐I = 13, *S* = 17).

In the derivation cohort, all nmCD63‐containing combined models achieved significantly higher AUCs than did nmCD63 alone (all *p* < 0.05, Delong's test). The nmCD63 + SOFA model demonstrated the strongest discriminative ability (AUC, 0.943; 95% CI, 0.892–0.994), whereas the nmCD63 + CRP + PCT model achieved the highest overall accuracy (94.37%). None of the models showed evidence of severe multicollinearity (VIF < 3), and all except the nmCD63 + SOFA model demonstrated adequate goodness of fit (Hosmer–Lemeshow *p* > 0.05). The significant Hosmer–Lemeshow result for the nmCD63 + SOFA model is likely attributable to the small sample size (*n* = 71) and does not invalidate its excellent discriminative performance.

In the independent validation cohort, all combined models maintained their performance advantages over nmCD63 alone (all *p* < 0.05, Delong's test). The nmCD63 + SOFA and nmCD63 + CRP models achieved the highest overall accuracy (90.00%), with AUCs of 0.919 (95% CI, 0.838–1.000) and 0.913 (95% CI, 0.831–0.995), respectively. No model showed evidence of overfitting as all performance metrics remained stable or improved when applied to the validation cohort using coefficients derived exclusively from the derivation cohort.

### 2.5 Dynamic Changes of Nuclear Membrane CD63 and Prognostic Stratification in Infected Patients

**FIGURE 4:**
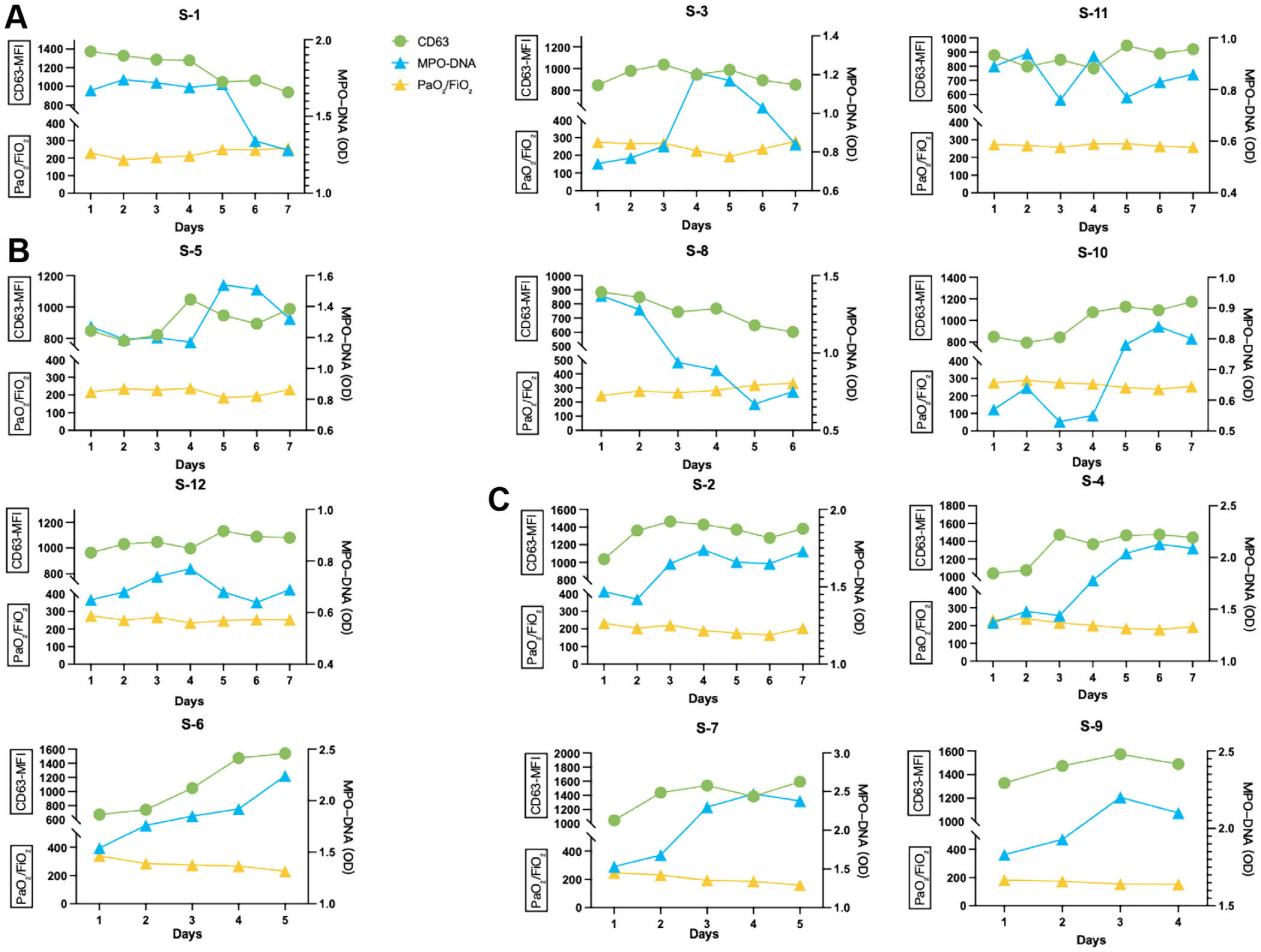
Longitudinal changes in nuclear membrane CD63, MPO‐DNA, and PaO_2_/FiO_2_ in individual patients with sepsis (S cohort). Longitudinal changes in nuclear membrane CD63, MPO‐DNA, and PaO_2_/FiO_2_ in individual patients with sepsis (S cohort). Patients were stratified according to 7‐day clinical outcomes: improved (A), stable (B), and progressive (C). (A) Patients with improved outcomes (transferred to general ward) showed a decreasing trend in nmCD63. (B) Patients with stable condition exhibited relatively stable nmCD63 levels. (C) Patients with progressive disease (including non‐survivors) showed a persistent upward trend with consistently high MFI levels. Changes in nuclear membrane CD63 in all three groups preceded alterations in plasma NETs and respiratory index.

To evaluate the prognostic value of nuclear membrane CD63 (nmCD63) over time, serial measurements were performed at baseline, Day 3, and Day 7 (days 1–7) in 12 patients with nonsevere pulmonary infection (NS‐I cohort) and 12 patients with pulmonary sepsis (S cohort). Clinical outcomes were graded as improved, stable, or progressive (higher scores indicated a worse status). Individual longitudinal changes in nmCD63, MPO‐DNA, and PaO_2_/FiO_2_ are presented in Figure S4A–C (NS‐I cohort) and Figure 4A–C (S cohort).

Spearman rank correlation was used to assess associations between nmCD63 measurements and clinical outcome (Table 3, Table S11). In the NS‑I cohort, baseline nmCD63 (*r*_s_ = −0.215, *p* = 0.503), day 3 nmCD63 (*r*_s_ = 0.138, *p* = 0.669), and ΔDay3 (*r*_s_ = 0.309, *p* = 0.328) showed no significant correlation with outcome. By contrast, day 7 nmCD63 (*r*_s_ = 0.799, *p* = 0.002) and ΔDay7 nmCD63 (*r*_s_ = 0.879, *p* < 0.001) were strongly positively associated with worse clinical outcomes. In the S cohort, baseline nmCD63 also lacked prognostic value (*r*_s_ = 0.232, *p* = 0.469). However, significant predictive capacity emerged as early as day 3: day 3 nmCD63 (*r*_s_ = 0.603, *p* = 0.038) and ΔDay3 (*r*_s_ = 0.782, *p* = 0.003) were both significantly correlated with outcome. Day 7 nmCD63 (*r*_s_ = 0.860, *p* < 0.001) and ΔDay7 nmCD63 (*r*_s_ = 0.853, *p* < 0.001) remained the strongest prognostic markers.

**TABLE 3:** Spearman correlation of nuclear membrane CD63 measurements with clinical outcome.

Marker	NS‐I cohort *r*_s_ (P)	S cohort *r*_s_ (P)
Baseline	−0.215 (0.503)	0.232 (0.469)
Day 3	0.138 (0.669)	0.603* (0.038)
Day 7	0.799** (0.002)	0.860*** (<0.001)
ΔDay3	0.309 (0.328)	0.782** (0.003)
ΔDay7	0.879*** (<0.001)	0.853*** (<0.001)

*Note*: Outcomes were coded as 1 = improved, 2 = stable, 3 = progressive (higher values indicate worse clinical status). Spearman rank correlation was used for nonparametric analysis. *p < 0.05, **p < 0.01, ***p < 0.001. ΔDay3 indicates day 3 value minus baseline value; ΔDay7 indicates day 7 value minus baseline value.

Abbreviations: NS‐I, no severe infection cohort; S, sepsis cohort.

Dynamic changes in nmCD63, particularly ΔDay7, provided robust prognostic information, whereas single early measurements demonstrated limited utility. The earlier emergence of predictive signals in the S cohort reflects more rapid immune and clinical deterioration in sepsis.

**FIGURE 5:**
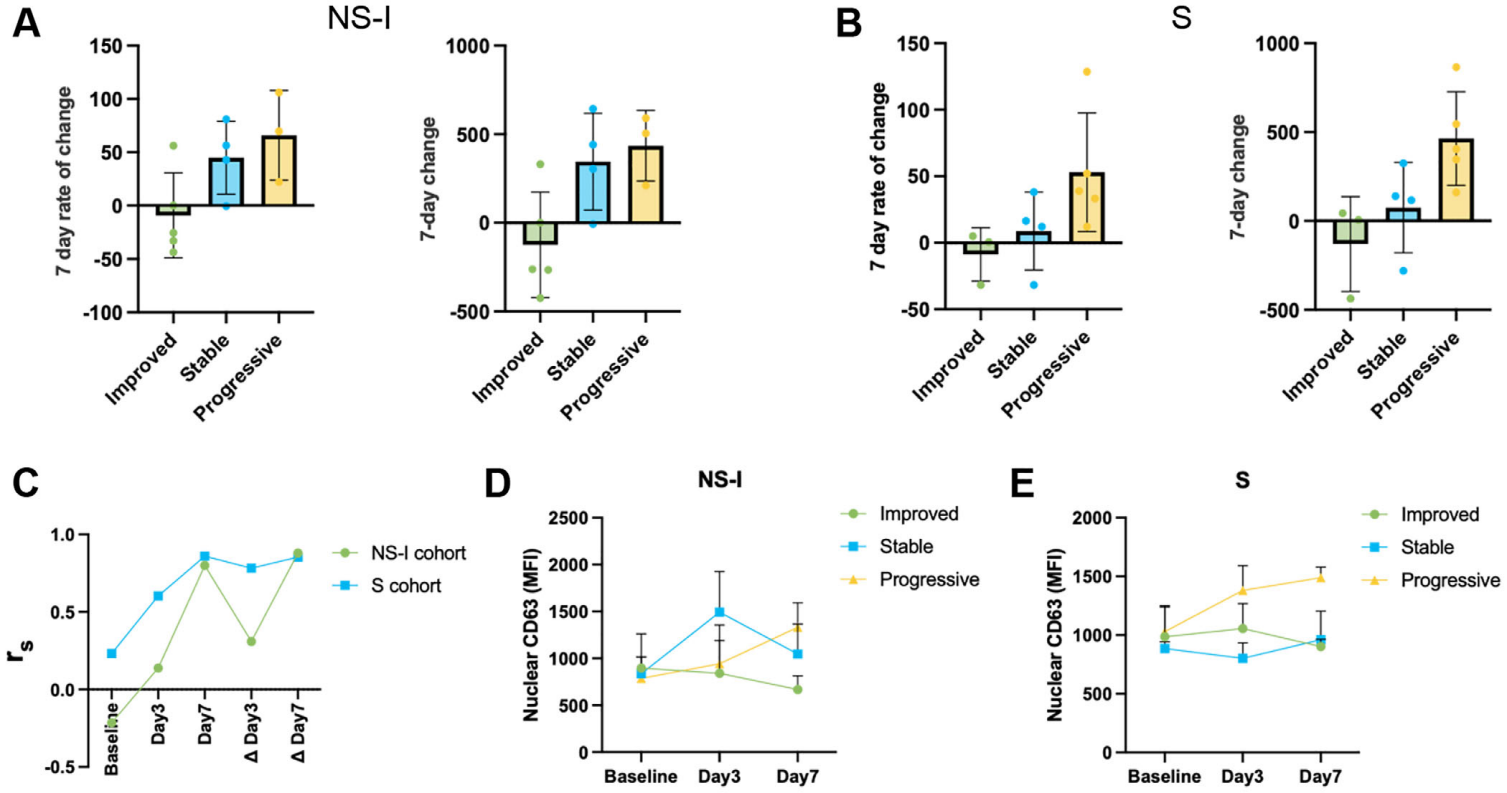
Prognostic stratification by dynamic trajectories of nuclear membrane CD63 in infected patients. (A) Percentage change in nuclear membrane CD63 from baseline to day 7 by clinical outcome in the NS‐I cohort. (B) Percentage change in nuclear membrane CD63 by clinical outcome in the S cohort. (C) Mean dynamic trajectories of nuclear membrane CD63 in the NS‐I cohort. (D) Mean dynamic trajectories in the S cohort. (E) Overlay of mean trajectories across outcome groups. Improved outcomes were associated with decreasing nuclear membrane CD63 levels, whereas progressive disease was associated with increasing trajectories.

Patients were stratified into three trajectory groups based on ΔDay7%: decreasing (< −20%), stable (−20% to 20%), and increasing (> 20%). These thresholds were predefined to maximize differentiation across the three clinical outcome tiers (improved, stable, and progressive), consistent with the conventional criteria for clinically meaningful biomarker changes. The outcome distributions are shown in Figure 5 and Tables S12 and S13. In the NS‐I cohort, the outcome distribution differed significantly across the groups (*p* = 0.023, Fisher's exact test). All patients in the decreasing group (100.0%) had improved outcomes, whereas 57.1% of the patients in the increasing group developed progressive disease. None of the patients in the decreasing or stable groups experienced clinical worsening. In the S cohort, prognostic stratification was even more pronounced (*p* = 0.005). All patients in the decreasing group (100.0%) improved, and 80.0% of those in the increasing group progressed. No progressive outcomes were also observed in either the decreasing or stable groups.

The mean trajectory curves for nmCD63 by outcome group are presented in Figure 5A–E. Patients with progressive disease showed continuously increasing nmCD63 levels, whereas those with improved outcomes exhibited a marked decline, confirming that nmCD63 trajectories accurately reflect clinical disease course.

## 3 Discussion

Sepsis remains the leading cause of mortality in ICUs worldwide, with its core clinical dilemma being the inability to distinguish early infection from noninfectious inflammation and the lag in predicting disease progression [12, 13]. Current biomarkers, including CRP, PCT, and the SOFA score, fail to accurately capture the central pathophysiological hallmark of sepsis initiation: early abnormal neutrophil activation [14, 15]. Our team has previously identified targeted nuclear degranulation as a critical upstream event in NET formation. Based on this mechanism, we conducted the first systematic validation of nmCD63 as a surrogate marker for targeted nuclear degranulation in a two‐center prospective cohort of 256 participants. nmCD63 not only significantly outperformed conventional biomarkers in early infection discrimination but also provided an advance warning of disease progression through dynamic monitoring, offering a novel tool for the precise stratified management of sepsis.

In clinical practice, differentiating noninfectious systemic inflammatory response syndrome from early bacterial infection is the cornerstone of rational antibiotic use and remains one of the most challenging problems in critical care medicine [9]. Our results showed that nmCD63 achieved an AUC of 0.794 for distinguishing NI from NS‐I patients, which was significantly superior to CRP (0.536) and PCT (0.628). This advantage has a clear pathophysiological basis; targeted nuclear degranulation is among the earliest events in neutrophil activation following pathogen exposure, whereas the hepatic acute‐phase response reflected by CRP and PCT typically lags by 6–12 h, and the SOFA score only assesses established organ dysfunction [16, 17, 18, 19].

nmCD63 demonstrated highly consistent diagnostic performance across two independent centers and achieved a performance comparable to that of the gold‐standard biomarker CRP (AUC, 0.8989 vs. 0.9063), while outperforming PCT for sepsis identification (NS‐I vs. S). Unlike conventional biomarkers that reflect non‐specific systemic inflammation, nmCD63 directly measures neutrophil activation status, which explains its strong correlation with the SOFA score (*r* = 0.82 in the derivation cohort, *r* = 0.80 in the validation cohort). The high negative predictive value of nmCD63 is most clinically useful in diagnostically ambiguous scenarios such as postoperative fever with no identifiable infectious focus, trauma patients with systemic inflammation driven by tissue injury rather than infection, and ICU patients with mildly elevated inflammatory markers where bacterial involvement is uncertain. In these settings, a negative nmCD63 result provides clinicians with greater confidence that significant pathogen‐driven neutrophil activation does not occur.

However, nmCD63 should not be used as a standalone justification for withholding or delaying antimicrobial therapy. Clinical judgment, microbiological culture results, imaging findings, and the overall clinical trajectory remain the primary determinants of treatment decisions. For antimicrobial stewardship programs, nmCD63 can support risk stratification, and patients with persistently negative or declining nmCD63 levels are candidates for closer observation while cultures are pending, rather than immediate initiation of broad‐spectrum empiric therapy, whereas patients with persistently rising nmCD63 levels should remain on full antimicrobial coverage until clear clinical improvement is documented.

The groundbreaking finding of this study was that changes in nmCD63 levels consistently preceded alterations in all conventional clinical indicators. Serial 7‐day monitoring revealed that increases or decreases in nmCD63 occurred earlier than changes in circulating NETs (MPO‐DNA), oxygenation index (FiO_2_), and CRP and PCT levels in both non‐septic and septic patients. This unique kinetic signature makes nmCD63 a true “predictive” biomarker rather than a “retrospective” diagnostic tool.

Stratification analysis based on 7‐day nmCD63 trajectories further reinforced its clinical utility; all patients with a decreasing nmCD63 trajectory achieved clinical improvement, whereas 57.1% of non‐septic patients and 80.0% of septic patients with an increasing trajectory experienced disease progression. Critically, none of the patients in the decreasing or stable trajectory groups developed progressive disease. This finding provides a clear actionable standard for clinical decision‐making. Patients with persistently elevated nmCD63 levels require prompt treatment escalation and enhanced organ support, whereas those with declining nmCD63 levels may be candidates for de‐escalation to avoid overtreatment. By contrast, changes in CRP and PCT levels consistently lagged behind clinical deterioration and failed to provide an effective early warning.

To further optimize the diagnostic performance, we constructed and externally validated combined models integrating nmCD63 with established clinical and laboratory markers. All nmCD63‐containing combined models significantly outperformed single biomarkers in both the derivation and validation cohorts, with the nmCD63 + SOFA model demonstrating the strongest discriminative ability (AUC = 0.943 in the derivation cohort and AUC = 0.919 in the validation cohort). DCA further verified its practical clinical value; the nmCD63 + SOFA model delivered the highest net benefit across the 0.2–0.8 threshold probability range, which matched the threshold range typically referenced by clinicians when deciding to initiate, escalate, or de‐escalate sepsis treatment.

Application of this model in routine clinical practice is straightforward. For any critically ill patient with suspected infection, clinicians first calculate the SOFA score using standard vital signs and routine laboratory data, then obtain the nmCD63 measurement and input both into the pre‐specified logistic regression equation to generate a sepsis probability. A high probability prompts closer monitoring and earlier escalation of organ support, whereas a low probability makes clinically significant sepsis substantially less likely. Because the model requires only a standard flow cytometer and the same laboratory tests already used for SOFA scoring, it is compatible with most tertiary care ICUs, including many resource‐limited settings where flow cytometry is already available for routine hematology testing.

Importantly, all combined models were validated using regression coefficients derived exclusively from the derivation cohort without any refitting, effectively avoiding overfitting and demonstrating their generalizability and stability. Thus, integrating nmCD63 into existing clinical risk stratification tools could significantly improve the accuracy of sepsis diagnosis and inform individualized treatment strategies.

Our clinical findings are consistent with the emerging paradigm that targeted nuclear degranulation is a key upstream event in NET formation [5, 6, 7]. We observed a stepwise increase in nmCD63 expression with increasing disease severity, accompanied by a reciprocal decrease in cytoplasmic MPO and an increase in nuclear MPO, providing direct clinical evidence of azurophilic granule‐nuclear membrane fusion in human sepsis. This process delivers granular enzymes such as MPO and neutrophil elastase into the nucleus, mediating chromatin decondensation and NET release. Excessive NET formation is a central driver of sepsis‐induced multiple organ injury [20, 21].

Compared to existing sepsis biomarkers, the core innovation of nmCD63 lies in its mechanism‐driven design. Conventional biomarkers are mostly downstream products of the inflammatory response and lack disease specificity, whereas nmCD63 directly measures the center of sepsis pathogenesis and abnormal neutrophil activation, thus offering higher specificity and an earlier diagnostic window. This design paradigm also provides a reference for the development of biomarkers of other inflammatory diseases.

In addition to classic inflammatory biomarkers, nmCD63 has clear advantages over emerging NET‐associated indicators, including circulating MPO‐DNA complexes, citrullinated histone H3 (CitH3), and cell‐free DNA. These circulating NET markers only capture fully released extracellular NETs, a late downstream event that lags hours after initial neutrophil activation. They also suffer from poor analytical stability because extracellular NET fragments are rapidly degraded by circulating nucleases, leading to underestimated results and high inter‐assay variability [4, 22]. By contrast, nmCD63 measures targeted nuclear degranulation, the rate‐limiting step upstream of NET formation, and provides an earlier diagnostic window than circulating NET biomarkers. The detection of purified intact nuclei also avoids nuclease degradation, ensuring a more stable and reproducible quantification.

A major strength of this study is its strict, two‐center, independent external validation design. Unlike single‐center studies that used internal random splitting for validation, our validation cohort was recruited from a geographically distinct region in southern China with a different patient population and clinical practice pattern from the derivation cohort in northern China. The consistent performance of nmCD63 and all combined models across both cohorts without any model adjustment strongly supports the robustness and cross‐regional generalizability of our findings.

Overall, nmCD63 not only exhibited stepwise elevation with disease severity and superior diagnostic performance for early infection and sepsis but also provided unique dynamic prognostic information through serial monitoring. To systematically compare the comprehensive clinical utility of nmCD63 with that of conventional sepsis biomarkers, their core characteristics and performance metrics are summarized in Table S14.

This study has several limitations. First, this was a two‐center prospective study conducted exclusively in mainland China, with participants recruited from two tertiary academic hospitals. While our findings were consistent across independent derivation and validation cohorts, validation in multiethnic populations and different healthcare systems is required to confirm the global generalizability of the nmCD63 thresholds. Second, the dynamic monitoring subgroup was restricted to 12 patients per cohort (NS‐I and S) with pulmonary infections. This small sample size limits the statistical power for detecting smaller effect sizes; therefore, these trajectory findings should be considered hypothesis generating rather than confirmatory. Neutrophil activation kinetics and disease trajectories vary across infection sites, such as the intra‐abdominal region, bloodstream, urinary tract, skin, and soft tissue. Thus, the 7‐day nmCD63 trajectory prognostic findings cannot be directly extrapolated to nonpulmonary infections, and further studies with diverse primary infection sites are required to verify their broader applicability. Third, the current nmCD63 detection workflow relies on isolated neutrophils and purified nuclei, which involves multiple centrifugation and density gradient separation steps. This complex operation prolongs testing time and restricts routine bedside clinical use. In future translational research, we plan to develop a simplified direct whole‐blood flow cytometry detection method that eliminates the need for nuclear purification. This optimized rapid assay will shorten the processing time and lower technical thresholds, facilitating wider clinical promotion of nmCD63.

## 4 Conclusions

This study translated the basic immunological discovery of targeted nuclear degranulation into the clinically measurable biomarker, nmCD63. nmCD63 outperformed conventional biomarkers in early infection discrimination and provided an advanced warning of disease progression through serial monitoring, and nmCD63‐based combined models further improved the net clinical benefit. These findings have practical implications in patient care. In the emergency department, nmCD63 can help frontline clinicians quickly triage patients with suspected infection, and its high negative predictive value indicates that a normal result effectively rules out significant bacterial involvement, which is especially useful when clinical signs are ambiguous. In the ICU, daily nmCD63 monitoring provides clinicians an early heads‐up on which patients are heading toward deterioration, days before SOFA scores or conventional biomarkers start trending incorrectly. This early signal could allow for timely adjustments to supportive care, fluid management, and organ support. For risk stratification, 7‐day nmCD63 trajectories can help identify patients who are likely to improve versus those at the highest risk of progression, supporting a more personalized allocation of monitoring intensity and resources.

Larger multicenter studies are needed to confirm these findings and test whether nmCD63‐guided management translates to better patient outcomes.

## 5 Materials and Methods

### 5.1 Study Design and Population

This was a two‐center prospective cohort study with independent external validation conducted between August 2023 and November 2025. To rigorously evaluate the generalizability of nmCD63 as a clinical biomarker, we adopted a prespecified derivation‐validation design.

The derivation cohort was enrolled at the Jining Medical University Affiliated Hospital (*n* = 180). This cohort was used to optimize the flow cytometric assay for nmCD63, determine the optimal diagnostic cutoff values, and construct nmCD63‐based combined diagnostic models.

The independent external validation cohort comprised patients from the First Affiliated Hospital of Guangxi Medical University (*n* = 76). This cohort was independent from the derivation cohort in terms of patient recruitment, sample collection, laboratory testing, and clinical assessments. To minimize overfitting, all parameters derived from the derivation cohort were directly applied to this cohort without any recalibration, refitting, or threshold adjustment.

All eligible patients admitted to the participating ICUs during the study period were consecutively enrolled. A total of 256 participants were enrolled: 120 healthy volunteers (healthy control group, HC) and 136 adult critically ill patients (aged ≥ 18 years) with ICU stay > 24 h. Critically ill patients were further stratified into three groups per infection status and Sepsis‐3 criteria: non‐infectious critically ill group (NI, *n* = 35), non‐septic infection group (NS‐I, *n* = 44), and sepsis group (S, *n* = 57). Blood samples for nmCD63 measurement were obtained within 24 h of ICU admission (median, 6 h; IQR, 3–12 h). Among critically ill patients, 78.7% (*n* = 107) had received empiric antimicrobial therapy before sampling, at a median interval of 3 h (IQR, 1–8 h) from the first antibiotic dose.

Inclusion and exclusion criteria were as follows: The HC group comprised healthy volunteers aged ≥ 18 years and without acute inflammatory/infectious diseases, hematological disorders, malignancies, autoimmune diseases, genetic disorders, or HIV/syphilis infection within 1 month prior to enrolment. Pregnant and lactating women were excluded from this study.

Critically ill patients were categorized as aged ≥18 years and admitted to ICU with expected stay > 24 h. The exclusion criteria included hematological diseases, active malignancies, autoimmune disorders, HIV/syphilis infection, pregnancy/lactation, and immunosuppressive therapy within 30 days before enrolment.

The final cohort comprised 256 participants (HC = 120, NI = 35, NS‐I = 44, S = 57), including 180 in the derivation cohort and 76 in the independent validation cohort; a 24‐patient subgroup (12 NS‐I, 12 sepsis) underwent 7‐day dynamic monitoring. Sample size was determined by enrolment feasibility, as this was an exploratory biomarker study with no formal power calculation; the achieved sample was considered adequate for proof‐of‐concept given anticipated AUCs ≥ 0.80. Infection status was adjudicated by ICU physicians via integrated clinical, microbiological, and radiological assessment rather than culture alone to avoid misclassifying culture‐negative sepsis.

### 5.2 Flow Cytometric Detection of Nuclear Membrane CD63

Neutrophil nuclei were isolated by nitrogen cavitation, followed by OptiPrep density gradient purification. Briefly, neutrophils were purified by negative selection and resuspended in a hypotonic buffer. The cells (5 × 10^7^) were lysed at 380 psi for 5 min in a nitrogen cavitation bomb (Parr Instruments), followed by three washes at 500 × *g* to remove the cytosol and granules [23]. Nuclei were further purified on a discontinuous OptiPrep gradient (20%, 30%, and 40%) by centrifugation at 25,000 *g* for 25 min (no brake). The nuclear band at the 30%–40% interface was collected, washed, and resuspended in the assay buffer. This purification process removed contaminating plasma membrane fragments and unbound cytoplasmic granules, eliminated CD63 interference, and ensured assay specificity. Nuclear integrity was confirmed using SYTOX Green exclusion system.

Isolated nuclei were stained with PE‐conjugated anti‐CD63 antibody (PE, BioLegend, 353010) for 20 min at room temperature in the dark, washed with PBS, and analyzed on a BD FACSCanto II flow cytometer. The gating strategy included the sequential selection of intact nuclei (FSC‐A/SSC‐A) and doublet exclusion (FSC‐A/FSC‐H), with CD63 expression quantified as median fluorescence intensity (MFI) using FlowJo v10.8. The PE voltage was optimized with controls and fixed throughout the study, and the instrument performance was monitored monthly using CS&T beads. Only nuclear preparations with more than 90% integrity (SYTOX Green exclusion) were used for staining. Representative validation images are provided in Figure S2. All flow cytometric measurements of nmCD63 were performed by laboratory personnel who were blinded to the clinical classification (NI, NS‐I, or sepsis) and clinical outcomes of the participants. The attending ICU physicians who determined the infection status and Sepsis‐3 classification were also blinded to the nmCD63 results at the time of clinical assessment. Clinical data and laboratory results (CRP, PCT, SOFA) used for model construction were retrieved from the electronic medical record by independent researchers not involved in sample processing. No indeterminate nmCD63 results were encountered, as nmCD63 was quantified as a continuous variable (median fluorescence intensity) without a predefined equivocal range. During nuclear preparation, approximately 6% of isolates (15 of 256 preparations) failed to meet the > 90% integrity threshold by SYTOX Green exclusion and were discarded; these samples were reprocessed from remaining whole‐blood aliquots, and all participants ultimately yielded valid nmCD63 measurements with no missing data. The rate of failed preparation was balanced across clinical groups (NI, 5.7%; NS‐I, 6.8%; S, 7.0%; HC, 5.0%; *p* = 0.96) and was unrelated to disease severity or sample storage duration. No study‐related adverse events were recorded; all procedures involved only standard peripheral venous blood sampling.

### 5.3 ROC Curve Analysis and DCA

The diagnostic performance of nuclear membrane CD63, conventional clinical biomarkers (CRP and PCT), and neutrophil parameters was evaluated via ROC curve analysis using GraphPad Prism 9.0.

### 5.4 Statistical Analysis

Continuous variables are presented as mean ± SD or median (IQR), with between‐group comparisons by one‐way ANOVA (Tukey's) or Kruskal–Wallis (Dunn's) test, and correlations by Pearson's or Spearman's coefficient. Diagnostic performance was evaluated by ROC curve analysis with Delong's test. Optimal cutoffs were determined exploratorily in the derivation cohort (Youden index) and applied to the validation cohort without refitting. Univariate logistic regression was performed for each predictor; variables with *p* < 0.10 were entered into multivariable models using backward stepwise selection (α_entry = 0.05, α_removal = 0.10), with all predictors as continuous variables. Linearity was confirmed by Box–Tidwell test (all *p* > 0.05); multicollinearity was assessed by VIF (< 3) and calibration by Hosmer–Lemeshow test. Analyses used GraphPad Prism 9.0 and SPSS 26.0 (two‐sided *p* < 0.05), with complete‐case analysis and no missing primary‐outcome or nmCD63 data.

## Author Contributions

**Xingxin Gao**: conceptualization, resources, writing – original draft, writing – review and editing. **Min Zhang**: conceptualization, validation, writing – original draft, writing – review and editing. **Linbin Li**: methodology, validation, formal analysis, data curation. **Haihua Lyu**: methodology, formal analysis, investigation. **Xucheng Wei**: methodology, formal analysis, investigation, data curation. **Dehui Li**: investigation. **Yiming Shao**: conceptualization, validation, resources, writing – original draft, writing – review and editing. All authors read and approved the final manuscript.

## Funding

This work was supported by the National Natural Science Foundation of China (82302800), the Guangxi Medical and Health Appropriate Technology Development and Promotion Application (S2022087), the Natural Science Foundation of Guangxi Zhuang Autonomous Region (2026GXNSFAA00640133), and the Postdoctoral Innovation Program in Shandong Province (SDCX‑ZG‑202400032). The funders had no role in the design of the study, collection, analysis, and interpretation of data, or in writing of the manuscript.

## Ethics Statement

This was a noninterventional observational biomarker study and was not registered in a clinical trial registry, as trial registration was not required by the institutional ethics committees for observational studies at the time of initiation. All study procedures were approved by the institutional ethics committees (2025‐S0838; 2023‐03‐B024) and conducted in accordance with the Declaration of Helsinki.

## Conflicts of Interest

The authors declare no conflicts of interest.

## Supporting information



Supporting information

## Data Availability

The datasets used and analyzed during the current study are available from the corresponding author upon reasonable request, subject to ethical and institutional regulations. All core data supporting the findings of this study are included within the article and its Supporting Information. The study protocol is available from the corresponding author upon reasonable request.
