# Supplementary material for: Dynamic Changes of Neutrophil Nuclear Membrane CD63 as a Potential Biomarker for Early Adjunctive Diagnosis and Prognostic Evaluation of Sepsis in Critically Ill Patients: A Prospective Cohort Study
Source: MedComm (2020). 2026 Sep 16;7(10):e71007. doi: 10.1002/mco2.71007 (PMC13583469; doi:10.1002/mco2.71007)
Supplement: Supplementary file 1 [file MCO2-7-e71007-s001.docx]

**Supplementary Information**

**Dynamic Changes of Neutrophil Nuclear Membrane CD63 as a Potential Biomarker for Early Adjunctive Diagnosis and Prognostic Evaluation of Sepsis in Critically Ill Patients: A Prospective Cohort Study**

**Running title:** Nuclear CD63 for Evaluating Sepsis

Xingxin Gao^1, #^, Min Zhang^2, #^, Linbin Li^1, #^, Haihua Lyu^1^, Zhihao Liu^3^, Xucheng Wei^1^, Dehui Li^1^, Yiming Shao^1, *^

^1^ Department of Burns Surgery, The First Affiliated Hospital of Guangxi Medical University, Nanning 530000, Guangxi, China.

^2^ Department of Critical Care Medicine, First Affiliated Hospital of Guangxi Medical University, Nanning 530000, Guangxi, China.

^3^ Department of Clinical Medicine, Jining Medical University，Jining 272004，Shandong, China

^*^ **Correspondence to:** Yiming Shao, PhD, Department of Burns Surgery, The First Affiliated Hospital of Guangxi Medical University, Nanning 530000, Guangxi, China. **Email:** symlcyb@163.com

^#^ X.G., M.Z. and L.L. contributed equally to this work.

Figure S1 Targeted nuclear degranulation occurs in neutrophils from sepsis patients. (A) Schematic diagram illustrating that following activation in sepsis, neutrophils undergo cytoskeletal rearrangement, with azurophilic granules trafficking toward the nucleus along the cytoskeleton and fusing with the nuclear membrane to release MPO and other mediators. These events promote intranuclear DNA unwinding and ultimately mediate the release of NETs. (B) Immunofluorescence showing increased colocalization of the azurophilic granule marker CD63 (green) with the nuclear membrane marker LaminB1 (red) in neutrophils from sepsis patients. (C) Immunofluorescence demonstrating enhanced intranuclear translocation of MPO (green) in neutrophils from sepsis patients. (D) Transmission electron microscopy revealing the morphological feature of “targeted nuclear degranulation” in neutrophils from sepsis patients. (E) Western blot analysis showing that in healthy neutrophils stimulated with PMA (100 ng/mL) in vitro, intranuclear MPO and nuclear membrane CD63 were gradually elevated, accompanied by partial disruption of LaminB1.

Figure S2 Flow cytometric detection of nuclear membrane CD63. (a) After nuclear extraction from neutrophils, intact nuclei were visualized by SYTOX Green staining, followed by staining with CD63 flow antibody and subsequent flow cytometric analysis (5×10^7^ cell). (b, c) Neutrophils were stimulated in vitro with LPS (1 μg/mL) or PMA (100 ng/mL) for 30 min, respectively. Nuclear extraction and flow cytometric detection revealed that both PMA and LPS significantly upregulated the expression of nuclear membrane CD63.

Figure S3 Study Design Flowchart. This flowchart shows the research design for analysing the nuclear membrane CD63 in peripheral blood PMNs. The left section depicts the healthy control group and the corresponding inclusion criteria, while the right section details the stratification protocol for ICU patients. Patients with clinically suspected infection were categorised into an infection group (I, n =101) and a non-infection group (NI, n = 35). In accordance with Sepsis-3 criteria, the I group was further subdivided into no severe infection cases (NS–I, n = 44) and sepsis cases (S, n = 57). Arrows illustrate the logical associations between infection severity and patient stratification. Five common infection a etiologies are listed at the lower left: pneumonia, bloodstream infections, catheter-related sepsis, intra-abdominal infections, urosepsis, and surgical site infections.

Figure S4 Longitudinal changes in nuclear membrane CD63, MPO‑DNA, and PaO_2_/FiO_2_ in individual patients with nonsevere infection (NS-I cohort). Patients were stratified according to 7-day outcomes: improved condition (A), stable condition (B), and progression to sepsis(C). (A) Nuclear membrane CD63 exhibited a decreasing trend in patients with improved condition. (B) Nuclear membrane CD63 also trended upward in patients with stable condition, but remained at a relatively low MFI level. (C) Nuclear membrane CD63 showed an increasing trend in patients progressing to sepsis. Changes in nuclear membrane CD63 in all three groups preceded alterations in plasma NETs and respiratory index.

**Table S1 Baseline characteristics of the derivation cohort**

| 1. **Variables** | **Healthy** | **NI** | **NS-I** | **Sepsis** | **P value** |
| --- | --- | --- | --- | --- | --- |
| Sample size (all) | 84 | 25 | 31 | 40 |  |
| Sex (male %) | 49(58.3%) | 16(60%) | 20(61.3%) | 27(55.0%) | >0.05 |
| Age (years) | 40.37±6.072 | 58.44±11.05 | 57.16±12.37 | 62.38±9.01 | <0.001 |
| BMI (kg/m^2^) | 20.67±1.01 | 22.62±3.034 | 22.90±3.136 | 23.18±3.87 | >0.05 |
| 2. Vital signs |  |  |  |  |  |
| Body temperature (°C) | 36.56±0.35 | 36.91±1.215 | 38.46±0.5959 | 39.18±0.60 | <0.001 |
| SBP (mmHg) | 121.6±12.48 | 122.8±20.22 | 119.9±20.51 | 117.60±24.40 | >0.05 |
| Heart rate (bpm) | 72.96±16.02 | 88.80±25.80 | 106.8±16.54 | 113.70±22.29 | <0.001 |
| 3. Primary site of infection |  |  |  |  |  |
| Lower respiratory tract | NA | NA | 15 | 19 | - |
| Intra-abdomen | NA | NA | 3 | 5 | - |
| Urinary system | NA | NA | 3 | 3 | - |
| Skin and soft tissue | NA | NA | 4 | 6 | - |
| Bloodstream | NA | NA | 6 | 7 | - |
| 4. Comorbidities |  |  |  |  |  |
| Hypertension | NA | 10 (41.7%) | 14(45.2%) | 16 (40.0%) | - |
| Diabetes mellitus | NA | 6 (25.0%) | 10 (32.2%) | 10 (25.0%) | - |
| CHD | NA | 0 (0.0%) | 11 (35.5%) | 14 (35.0%) | - |
| COPD | NA | 0 (0.0%) | 9 (29.0%) | 16(40.0%) | - |
| 5. Laboratory parameters |  |  |  |  |  |
| CRP(mg/L)  median(IQR) | NA | 33.42  (25.16-41.98) | 38.42 (21.44-43.99) | 89.39  (54.79-104.6) | <0.001 |
| PCT  median(IQR) | NA | 1.370 (0.40-4.83) | 2.910 (1.530-5.420) | 7.745 (5.590-9.665) | <0.001 |
| NC (10^9^ /L) | 6.590±1.40 | 8.71±3.37 | 9.799±2.551 | 12.32±4.699 | <0.001 |
| NC(%) | 55.77±11.03 | 81.25±5.650 | 83.42±4.784 | 88.38±5.795 | <0.001 |
| 6. Core study biomarkers |  |  |  |  |  |
| Nuclear membrane CD63/10^7^cell（MFI） | 237.2±53.59 | 392.4±181.10 | 680.3±301.40 | 1350±381.89 | <0.001 |
| MPO in nucleus (ng/ml)/10^6^cell | 12.98±2.14 | 23.54±9.63 | 29.23±8.48 | 48.92±10.80 | <0.001 |
| MPO in cytoplasm (ng/ml)/10^6^cell | 542.7±94.49 | 450.9±66.73 | 344.0±100.41 | 179.9±117.61 | <0.001 |
| SOFA  median (IQR) | NA | 6.00  (5.00-7.00) | 5.00 (4.00-6.00) | 8.00  (7.00-9.00) | <0.001 |

Note: This table presents baseline characteristics of the derivation cohort enrolled at Jining Medical University Affiliated Hospital (August 2023 to December 2024, n=180). Continuous variables are presented as mean ± SD for normally distributed data or median (IQR) for non-normally distributed data. Categorical variables are presented as number (percentage). Between-group comparisons were performed using one-way ANOVA, Kruskal-Wallis test, or chi-square test as appropriate. P values reflect overall comparisons across the four groups.

**Table S2 Baseline characteristics of the validation cohort**

| 1. **Variables** | **Healthy** | **NI** | **NS-I** | **Sepsis** | **P value** |
| --- | --- | --- | --- | --- | --- |
| Sample size (all) | 36 | 10 | 13 | 17 |  |
| Sex (male %) | 20(55.6%) | 7(70%) | 8(61.5%) | 12(70%) | >0.05 |
| Age (years) | 41.64±5.99 | 58.60±10.46 | 61.62±12.31 | 63.53±6.206 | <0.001 |
| BMI (kg/m2) | 20.88±0.97 | 22.84±3.05 | 23.88±3.273 | 23.688±2.811 | >0.05 |
| 2. Vital signs |  |  |  |  |  |
| Body temperature (°C) | 36.45±0.23 | 36.99±1.13 | 38.62±0.6309 | 39.27±0.49 | <0.001 |
| SBP (mmHg) | 124.2±12.20 | 125.1±18.22 | 126.7±20.39 | 120.5±18.02 | >0.05 |
| Heart rate (bpm) | 76.69±14.46 | 90.70±23.30 | 114.2±14.82 | 117.2±18.05 | <0.001 |
| 3. Primary site of infection |  |  |  |  |  |
| Lower respiratory tract | NA | NA | 7 | 9 | - |
| Intra-abdomen | NA | NA | 2 | 2 | - |
| Urinary system | NA | NA | 1 | 1 | - |
| Skin and soft tissue | NA | NA | 1 | 2 | - |
| Bloodstream | NA | NA | 2 | 3 | - |
| 4. Comorbidities |  |  |  |  |  |
| Hypertension | NA | 4(36.4%) | 7(53.8%) | 8(47.1%) | - |
| Diabetes mellitus | NA | 6 (54.5%) | 6 (46.2%) | 4(23.5%) | - |
| CHD | NA | 0 (0.0%) | 6 (46.2%) | 5 (29.4%) | - |
| COPD | NA | 0 (0.0%) | 4(30.8%) | 6(35.3%) | - |
| 5. Laboratory parameters |  |  |  |  |  |
| CRP(mg/L)  median(IQR) | NA | 34.47  (29.43-39.17) | 48.22  (45.68-57.56) | 71.40  (58.63-93.79) | <0.001 |
| PCT  median(IQR) | NA | 1.22  (0.30-4.98) | 4.00  (2.95-7.04) | 8.940  (7.22-10.51) | <0.001 |
| NC (109 /L) | 6.24±1.44 | 9.09±3.21 | 9.89±3.06 | 12.09±5.05 | <0.001 |
| NC(%) | 58.06±9.60 | 81.50±6.79 | 82.98±6.66 | 89.38±5.32 | <0.001 |
| 6. Core study biomarkers |  |  |  |  |  |
| Nuclear membrane CD63/107cell（MFI） | 253.0±88.83 | 439.7±215.50 | 742.0±242.8 | 1319±398.90 | <0.001 |
| MPO in nucleus (ng/ml)/106cell | 12.83±2.034 | 21.69±8.866 | 34.88±11.44 | 48.61±9.49 | <0.001 |
| MPO in cytoplasm (ng/ml)/106cell | 520.0±75.43 | 446.1±68.35 | 327.0±106.70 | 163.5±72.55 | <0.001 |
| SOFA  median (IQR) | NA | 6.00  (6.00-7.00) | 5.00  (4.00-6.00) | 8.00  (7.00-10.00) | <0.001 |

Note: This table presents baseline characteristics of the independent external validation cohort enrolled at The First Affiliated Hospital of Guangxi Medical University (January 2025 to November 2025, n=76). Continuous variables are presented as mean ± SD for normally distributed data or median (IQR) for non-normally distributed data. Categorical variables are presented as number (percentage). Between-group comparisons were performed using one-way ANOVA, Kruskal-Wallis test, or chi-square test as appropriate. P values reflect overall comparisons across the four groups. No significant differences were observed in any demographic, clinical, or laboratory parameters between the derivation and validation cohorts (all P > 0.05), confirming their comparability for external validation.

**Table S3 Spearman rank correlation analysis between age and nmCD63 levels across all study groups**

| **Group** | **Spearman r** | **95% Confidence Interval** | **Two-tailed P value** | **Significance (α = 0.05)** |
| --- | --- | --- | --- | --- |
| Healthy | −0.1235 | −0.3011 to 0.0623 | 0.1788 | Not significant |
| NI | 0.0177 | −0.3266 to 0.3578 | 0.9197 | Not significant |
| NS-I | −0.2330 | −0.5024 to 0.0777 | 0.1280 | Not significant |
| S | −0.1281 | −0.3829 to 0.1448 | 0.3424 | Not significant |

Note: Spearman rank correlation was used to assess the association between age and nmCD63 levels in each subgroup. All analyses were two-tailed.

**Table S4 Area Under the Receiver Operating Characteristic Curve for Nuclear Membrane CD63, CRP, and PCT**

| **Diagnostic Task** | **Biomarker** | **Derivation Cohort AUC**  **(95% CI)** | **Validation Cohort AUC**  **(95% CI)** |
| --- | --- | --- | --- |
| Noninfected vs Nonsevere Infected | Nuclear membrane CD63 | 0.794 (0.676-0.913) | 0.907 (0.835-0.979) |
|  | C-reactive protein | 0.536 (0.382-0.691) | 0.926 (0.868-0.984) |
|  | Procalcitonin | 0.628 (0.467-0.789) | 0.858 (0.767-0.948) |
| Nonsevere Infected vs Septic | Nuclear membrane CD63 | 0.899 (0.831-0.967) | 0.877 (0.789-0.964) |
|  | C-reactive protein | 0.906 (0.842-0.970) | 0.886 (0.802-0.970) |
|  | Procalcitonin | 0.847 (0.765-0.930) | 0.814 (0.712-0.916) |

Note. AUC indicates area under the receiver operating characteristic curve. All cutoff values were pre-defined in the derivation cohort and applied to the validation cohort without recalibration; CI, confidence interval; CRP, C-reactive protein; PCT, procalcitonin.

# Table S5 Diagnostic Performance of Single Biomarkers for Distinguishing Noninfected vs Nonsevere Infected Patients

| **Biomarker** | **Cohort** | **AUC (95%CI)** | **Optimal Cutoff** | **Sensitivity (%)** | **Specificity (%)** | **PPV (%)** | **NPV (%)** | **Accuracy (%)** | **Youden Index** | **P value** |
| --- | --- | --- | --- | --- | --- | --- | --- | --- | --- | --- |
| Nuclear membrane CD63 | Derivation | 0.794 (0.676–0.913) | 323.5 (MFI) | 96.77 | 52 | 71.43 | 92.86 | 76.79 | 0.488 | <0.000001 |
| Nuclear membrane CD63 | Validation | 0.907 (0.835–0.979) | 323.5 (MFI)¹ | 95 | 77.42 | 84.44 | 92.31 | 87.32 | 0.724 | <0.000001 |
| CRP | Derivation | 0.536 (0.382–0.691) | 42.84 (mg/L) | 38.71 | 84 | 75 | 52.5 | 58.93 | 0.227 | 0.323449 |
| CRP | Validation | 0.926 (0.868–0.984) | 42.84 (mg/L)¹ | 35 | 82.31 | 74.22 | 52.21 | 57.12 | 0.244 | <0.000001 |
| PCT | Derivation | 0.628 (0.467–0.789) | 1.385 (ng/mL) | 87.1 | 52 | 69.23 | 76.47 | 71.43 | 0.391 | 0.059911 |
| PCT | Validation | 0.858 (0.767–0.948) | 1.385 (ng/mL)¹ | 95 | 70.97 | 80.85 | 91.67 | 84.51 | 0.66 | <0.000001 |

(NI vs NS-I, Derivation cohort n=56; Validation cohort n=23), Cutoff values were determined exploratorily in the derivation cohort using the Youden index and applied to the validation cohort without recalibration.

Notes:

1. Critical annotation: ¹The optimal cutoff value was uniformly adopted from the derivation cohort (no re-calculation in the validation cohort), which strictly follows the standard for external validation studies and avoids overfitting.
2. Units: MFI, median fluorescence intensity; CRP, C-reactive protein (mg/L); PCT, procalcitonin (ng/mL).

**Table S6 Delong Test Results: Pairwise Comparison of AUCs Among Biomarkers (Derivation vs Validation Cohorts)**

| **Comparison** | **Cohort** | **AUC Difference** | **Z-value** | **P value** | **Statistical Significance** | **Key Interpretation** |
| --- | --- | --- | --- | --- | --- | --- |
| Nuclear membrane CD63 vs CRP | Derivation | 0.2581 | 5.77 | <0.001 | Highly significant | Nuclear membrane CD63 shows significantly better diagnostic performance than CRP in the derivation cohort |
| Nuclear membrane CD63 vs CRP | Validation | 0.019 | 0.63 | 0.531 | Not significant | The diagnostic performance difference between CD63 and CRP is eliminated in the validation cohort, but CD63 maintains stable efficacy |
| Nuclear membrane CD63 vs PCT | Derivation | 0.1665 | 3.72 | 0.0002 | Highly significant | Nuclear membrane CD63 exhibits significantly superior efficacy compared to PCT in the derivation cohort |
| Nuclear membrane CD63 vs PCT | Validation | 0.0492 | 1.3 | 0.195 | Not significant | The efficacy advantage of CD63 over PCT is not statistically significant in the validation cohort, but CD63 still shows numerical superiority |
| CRP vs PCT | Derivation | 0.0916 | 2.05 | 0.0405 | Significant | CRP has marginally better diagnostic performance than PCT in the derivation cohort |
| CRP vs PCT | Validation | 0.0681 | 1.85 | 0.065 | Not significant (trend) | The performance difference between CRP and PCT is reduced to a non-significant trend in the validation cohort |

(NI vs NS-I, Derivation cohort n=56; Validation cohort n=23), Cutoff values were determined exploratorily in the derivation cohort using the Youden index and applied to the validation cohort without recalibration.

Notes:

1. Abbreviations: AUC, area under the receiver operating characteristic curve; NI, non-infectious critically ill patients; NS-I, non-septic infected patients.
2. Statistical method: Delong test was used for pairwise AUC comparisons (two-sided test). P < 0.05 was considered statistically significant.
3. Cohort composition: Derivation cohort included 25 NI and 31 NS-I patients; Validation cohort included 10 NI and 13 NS-I patients.

# **Table S7 Diagnostic Performance of Biomarkers for Sepsis Identification (NS-I vs S)**

| **Biomarker** | **Cohort** | **AUC (95%CI)** | **Optimal Cutoff¹** | **Sensitivity (%)** | **Specificity (%)** | **PPV (%)** | **NPV (%)** | **Accuracy (%)** | **Youden Index** | **P value** |
| --- | --- | --- | --- | --- | --- | --- | --- | --- | --- | --- |
| Nuclear membrane CD63 | Derivation | 0.899 (0.831–0.967) | 518.5 (MFI) | 85 | 80.65 | 82.93 | 83.08 | 83 | 0.656 | <0.000001 |
| Nuclear membrane CD63 | Validation | 0.877 (0.789–0.964) | 518.5 (MFI) | 82.35 | 76.92 | 79.17 | 80.77 | 80 | 0.593 | <0.000001 |
| CRP | Derivation | 0.906 (0.842–0.970) | 65.32 (mg/L) | 82.5 | 83.87 | 84.15 | 82.22 | 83.1 | 0.664 | <0.000001 |
| CRP | Validation | 0.886 (0.802–0.970) | 65.32 (mg/L) | 80 | 78.57 | 80 | 78.57 | 79.31 | 0.586 | <0.000001 |
| PCT | Derivation | 0.847 (0.765–0.930) | 2.85 (ng/mL) | 77.5 | 77.42 | 77.46 | 77.46 | 77.46 | 0.549 | <0.000001 |
| PCT | Validation | 0.814 (0.712–0.916) | 2.85 (ng/mL) | 76.47 | 73.33 | 75 | 74.81 | 74.81 | 0.498 | <0.000001 |

(NS-I vs S, Derivation cohort n=71; Validation cohort n=30)

Notes:

1. Critical standard: ¹Optimal cutoff values were derived solely from the derivation cohort and directly applied to the validation cohort without recalibration, in strict accordance with the external validation design.
2. Units: MFI, median fluorescence intensity; CRP, C-reactive protein (mg/L); PCT, procalcitonin (ng/mL).

# **Table S8. Delong Test Results: Pairwise Comparison of AUCs (NS-I vs S, Derivation vs Validation Cohorts)**

| **Comparison** | **Cohort** | **AUC Difference** | **Z-value** | **P value** | **Statistical Significance** | **Key Interpretation** |
| --- | --- | --- | --- | --- | --- | --- |
| Nuclear membrane CD63 vs CRP | Derivation | 0.0124 | 0.38 | 0.703 | Not significant | CD63 and CRP show comparable diagnostic efficacy for sepsis identification in the derivation cohort |
| Nuclear membrane CD63 vs CRP | Validation | 0.0089 | 0.25 | 0.802 | Not significant | Consistent efficacy between CD63 and CRP is maintained in the validation cohort |
| Nuclear membrane CD63 vs PCT | Derivation | 0.0517 | 1.62 | 0.105 | Not significant (trend) | CD63 shows numerical superiority over PCT, approaching statistical significance |
| Nuclear membrane CD63 vs PCT | Validation | 0.0632 | 1.89 | 0.059 | Not significant (trend) | The efficacy advantage of CD63 over PCT is retained in the validation cohort (trend-level significance) |
| CRP vs PCT | Derivation | 0.0393 | 1.21 | 0.226 | Not significant | No significant difference in diagnostic performance between CRP and PCT |
| CRP vs PCT | Validation | 0.0543 | 1.64 | 0.101 | Not significant (trend) | CRP shows slight numerical superiority over PCT in the validation cohort |

(NS-I vs S, Derivation cohort n=71; Validation cohort n=30)

Notes:

1. Abbreviations: NS-I, non-septic infected patients; S, septic patients; AUC, area under the receiver operating characteristic curve.
2. Statistical method: Delong test for pairwise AUC comparisons (two-sided test), P < 0.05 considered statistically significant.
3. Cohort composition: Derivation cohort included 31 NS-I and 40 S patients; Validation cohort included 13 NS-I and 17 S patients.
4. Clinical implication: For sepsis identification (NS-I vs S), CD63 demonstrates comparable efficacy to the gold-standard marker CRP, while showing consistent numerical superiority over PCT across both cohorts—supporting its utility as an alternative sepsis diagnostic marker.

**Table S9 Derivation Cohort (n = 71, Nonseptic Infected vs Septic Patients)**

| **Model** | **AUC (95% CI)** | **Maximum VIF** | **Hosmer-Lemeshow P Value** | **Complete Regression Equation (ln(p/1-p))** | **Variable Significance** |
| --- | --- | --- | --- | --- | --- |
| Single biomarkers |  |  |  |  |  |
| Nuclear membrane CD63 | 0.899 (0.831–0.967) | - | - | - | - |
| SOFA score | 0.898 (0.821–0.975) | - | - | - | - |
| C-reactive protein | 0.906 (0.842–0.970) | - | - | - | - |
| Procalcitonin | 0.847 (0.765–0.930) | - | - | - | - |
| Combined models |  |  |  |  |  |
| CD63 + SOFA | 0.943 (0.892–0.994) | 1.082 | 0.003 | -11.169 + 0.006×CD63 + 0.923×SOFA | CD63 (P < .001), SOFA (P = .001) |
| CD63 + CRP | 0.936 (0.882–0.990) | 1.293 | 0.972 | -9.427 + 0.004×CD63 + 0.097×CRP | CD63 (P = .002), CRP (P = .002) |
| CD63 + PCT | 0.929 (0.873–0.985) | 1.254 | 0.799 | -7.886 + 0.005×CD63 + 0.532×PCT | CD63 (P < .001), PCT (P = .001) |
| CD63 + CRP + PCT | 0.939 (0.886–0.992) | 1.462 | 0.952 | -17.253 + 0.005×CD63 + 0.131×CRP + 0.744×PCT | CD63 (P = .007), CRP (P = .018), PCT (P = .015) |

Linearity of the logit for all continuous predictors was confirmed using the Box-Tidwell test (all P > 0.05).

**Table S10 Validation Cohort (n = 30, Nonseptic Infected vs Septic Patients)**

| **Model** | **AUC (95% CI)** | **Validation Method** |
| --- | --- | --- |
| Single biomarkers |  |  |
| Nuclear membrane CD63 | 0.877 (0.789–0.964) | Direct application of derivation cohort cutoff |
| SOFA score | 0.865 (0.771–0.959) | Direct application of derivation cohort cutoff |
| C-reactive protein | 0.886 (0.802–0.970) | Direct application of derivation cohort cutoff |
| Procalcitonin | 0.814 (0.712–0.916) | Direct application of derivation cohort cutoff |
| Combined models |  |  |
| CD63 + SOFA | 0.919 (0.838–1.000) | Direct application of derivation cohort regression coefficients |
| CD63 + CRP | 0.913 (0.831–0.995) | Direct application of derivation cohort regression coefficients |
| CD63 + PCT | 0.906 (0.822–0.990) | Direct application of derivation cohort regression coefficients |
| CD63 + CRP + PCT | 0.916 (0.835–0.997) | Direct application of derivation cohort regression coefficients |

Linearity of the logit for all continuous predictors was confirmed using the Box-Tidwell test (all P > 0.05).

Notes:

1. Abbreviations: AUC, area under the receiver operating characteristic curve; CI, confidence interval; VIF, variance inflation factor; SOFA, Sequential Organ Failure Assessment; CRP, C-reactive protein; PCT, procalcitonin.

2. Statistical methods::Logistic regression models were constructed using backward stepwise selection (α<sub>entry</sub> = 0.05, α<sub>removal</sub> = 0.10).

VIF was used to assess multicollinearity; VIF < 3 indicates no severe multicollinearity.Hosmer-Lemeshow test was used to evaluate model goodness of fit; P > .05 indicates adequate fit.Delong test was used to compare AUC differences between combined models and nuclear membrane CD63 alone.

**Table S11 Spearman Rank Correlation Between CD63 Measurements and Clinical Outcome (S Cohort)**

| **CD63 Measurement** | **Spearman Correlation Coefficient (rₛ)** | **P-value** |
| --- | --- | --- |
| Baseline CD63 | 0.232 | 0.469 |
| Day 3 CD63 | 0.603* | 0.038 |
| Day 7 CD63 | 0.860** | <0.001 |
| ΔDay3 (Day 3 − Baseline) | 0.782** | 0.003 |
| ΔDay7 (Day 7 − Baseline) | 0.853** | <0.001 |

Note**:** Outcome was coded as 1=Improved, 2=Stable, 3=Progressive (higher values indicate worse clinical status); n=12. *p<0.05, **p<0.01. Spearman rank correlation was used for non-parametric analysis of ordered categorical outcomes. ΔDay3 = Day 3 nmCD63 − Baseline nmCD63; ΔDay7 = Day 7 nmCD63 − Baseline nmCD63.

**Table S12 Clinical Outcome Distribution by Nuclear Membrane CD63 Trajectory Groups**

| **Cohort** | **Trajectory Group** | **n** | **Improved, n (%)** | **Stable, n (%)** | **Progressive, n (%)** | **P** |
| --- | --- | --- | --- | --- | --- | --- |
| NS‑I | Decreasing | 3 | 3 (100.0) | 0 (0.0) | 0 (0.0) | 0.023 |
|  | Stable | 2 | 1 (50.0) | 1 (50.0) | 0 (0.0) |  |
|  | Increasing | 7 | 1 (14.3) | 2 (28.6) | 4 (57.1) |  |
| S | Decreasing | 2 | 2 (100.0) | 0 (0.0) | 0 (0.0) | 0.005 |
|  | Stable | 5 | 2 (40.0) | 3 (60.0) | 0 (0.0) |  |
|  | Increasing | 5 | 0 (0.0) | 1 (20.0) | 4 (80.0) |  |

**Note.** ΔDay7% = (day 7 value − baseline value)/baseline value × 100%. Trajectory groups were defined as decreasing (ΔDay7% < −20%), stable (−20% ≤ ΔDay7% ≤ 20%), and increasing (ΔDay7% > 20%). The ±20% ΔDay7% cutoffs were selected to maximize discrimination between different clinical outcomes. Fisher exact test was used to compare outcome distributions across groups. Data are presented as n (%). NS-I, no severe infection cohort; S, sepsis cohort.

## Table S13 Distribution of Clinical Outcomes According to CD63 Change Trajectories (S Cohort)

| **Trajectory Group** | **n** | **Improved, n (%)** | **Stable, n (%)** | **Progressive, n (%)** |
| --- | --- | --- | --- | --- |
| Decreasing (ΔDay7% < −20%) | 2 | 2 (100.0) | 0 (0.0) | 0 (0.0) |
| Stable (−20% ≤ ΔDay7% ≤ 20%) | 5 | 2 (40.0) | 3 (60.0) | 0 (0.0) |
| Increasing (ΔDay7% > 20%) | 5 | 0 (0.0) | 1 (20.0) | 4 (80.0) |
| P-value (Fisher’s exact) | — | — | — | 0.005 |

Note**:** ΔDay7% = (Day 7 nmCD63 − Baseline nmCD63)/Baseline nmCD63 × 100%. Trajectory groups were defined as: Decreasing (ΔDay7% < −20%), Stable (−20% ≤ ΔDay7% ≤ 20%), Increasing (ΔDay7% > 20%). Fisher’s exact test was used to compare outcome distributions across groups; n=12. Data are presented as n (%).

**Table S14 Comprehensive performance comparison of nmCD63 and conventional sepsis biomarkers**

| **Parameter** | **nmCD63** | **CRP** | **PCT** | **SOFA score** |
| --- | --- | --- | --- | --- |
| Mechanistic basis | Direct measure of neutrophil targeted nuclear degranulation (upstream initiating event of NET formation) | Hepatic acute-phase response (downstream systemic inflammation) | Hepatic acute-phase response (downstream systemic inflammation) | Assessment of established end-organ dysfunction |
| Diagnostic window | 0–6 h after neutrophil activation | 6–12 h after infection onset | 4–8 h after infection onset | ≥24 h after organ injury |
| **Early infection discrimination (NI vs NS-I)** |  |  |  |  |
| AUC (derivation cohort) | 0.794 | 0.536 | 0.628 | - |
| AUC (validation cohort) | 0.907 | 0.926 | 0.858 | - |
| Sensitivity (derivation) | >95% | 38.71% | 87.10% | - |
| Negative predictive value (derivation) | >92% | 52.50% | 76.47% | - |
| **Sepsis identification (NS-I vs S)** |  |  |  |  |
| AUC (derivation cohort) | 0.899 | 0.906 | 0.847 | - |
| AUC (validation cohort) | 0.877 | 0.886 | 0.814 | - |
| Combined model performance | nmCD63+SOFA: AUC 0.943 (derivation), 0.919 (validation) | - | - | - |
| Dynamic prognostic value | Excellent: 7-day trajectories predict outcome with 100% negative predictive value for disease progression; changes precede all conventional biomarkers by 24–48 h | Poor: changes lag clinical deterioration by 12–24 h | Moderate: delayed kinetic response | Moderate: reflects established organ damage, no predictive value |
| Specificity for infection | High (specific to neutrophil activation induced by pathogens) | Low (elevated in all inflammatory states) | Moderate (elevated in severe non-infectious inflammation) | Low (elevated in all organ dysfunction states) |
| Clinical utility | Early screening, rule-out infection, dynamic risk stratification, treatment guidance | Monitoring inflammation severity | Monitoring infection severity | Assessing organ dysfunction severity |

Notes: AUC, area under the receiver operating characteristic curve; CRP, C-reactive protein; NI, noninfected critically ill; NS-I, no severe infection; PCT, procalcitonin; S, sepsis; SOFA, Sequential Organ Failure Assessment; NET, neutrophil extracellular trap. All diagnostic parameters are from the derivation cohort unless otherwise specified.

**Materials and Methods**

**Infection and sepsis classification**

Infection was confirmed by attending ICU physicians via combined microbiological results, clinical manifestations and radiological findings, and categorised into five types per international consensus criteria: pneumonia, bloodstream infections, skin and soft tissue sepsis, intra-abdominal infections, and urosepsis.

Sepsis was diagnosed per the Sepsis-3 guidelines, defined as life-threatening organ dysfunction caused by dysregulated host response to infection, with a ≥2-point increase in Sequential Organ Failure Assessment (SOFA) score from baseline. Infected patients not meeting Sepsis-3 criteria were assigned to the NS-I group, and ICU patients without infection evidence were assigned to the NI group.All critically ill patients received standard ICU care per institutional protocols, including empiric antimicrobial therapy adjusted according to microbiological results, hemodynamic support, mechanical ventilation when indicated, and renal replacement therapy as needed. Treatment decisions were made by the attending ICU team independently of the study investigators. For the dynamic monitoring subgroup, all patients received guideline-concordant sepsis management including source control, appropriate antimicrobial therapy within 1 hour of recognition, and organ support as clinically indicated.

### Timing of Sampling and Antimicrobial Exposure

Peripheral blood samples for nmCD63 measurement were collected within 24 hours of ICU admission. The median time from ICU admission to blood sampling was 6 hours (IQR, 3–12). Among the 136 critically ill patients, 107 patients (78.7%) had received empiric antimicrobial therapy prior to blood sampling, with a median interval from first antibiotic dose to sampling of 3 hours (IQR, 1–8). The remaining 29 patients (21.3%) were sampled before initiation of antimicrobial therapy. Reference standard assessment (infection status and Sepsis-3 classification) was performed within 48 hours of admission, based on all available clinical, microbiological, and radiological data during that window.

**Neutrophil isolation**

Human peripheral blood neutrophils were isolated from EDTA-anticoagulated whole blood via negative selection using the EasySep™ Direct Human Neutrophil Isolation Kit (StemCell Technologies, #19666) per the manufacturer’s protocol. Purity of isolated neutrophils was validated by flow cytometry with CD66b-APC staining (BioLegend, #305118, Clone: G10F5, RRID:AB_2566607), and only samples with >95% purity were included for subsequent experiments.

**Immunofluorescence staining**

Isolated neutrophils were fixed with 4% paraformaldehyde, permeabilized with 0.2% Triton X-100, and blocked with 5% bovine serum albumin (BSA) for 1 h at room temperature. Cells were incubated overnight at 4°C with primary antibodies: anti-CD63 (Abcam, ab271286), anti-LaminB1 (Abcam, ab16048), and anti-myeloperoxidase (MPO, Abcam, ab208670). After washing, cells were incubated with corresponding Alexa Fluor-conjugated secondary antibodies for 1 h at room temperature in the dark, counterstained with DAPI, and imaged using a Zeiss LSM 880 laser scanning confocal microscope.

**Transmission electron microscopy**

Isolated neutrophils were fixed with 2.5% glutaraldehyde, post-fixed with 1% osmium tetroxide, dehydrated in graded ethanol series, and embedded in epoxy resin. Ultrathin sections (70 nm) were stained with uranyl acetate and lead citrate, and imaged using a Hitachi HT7800 transmission electron microscope to observe the morphological features of targeted nuclear degranulation.

**Western blot**

Neutrophils from healthy volunteers were stimulated with phorbol 12-myristate 13-acetate (PMA, 100 ng/mL) in vitro for indicated time points. Nuclear, cytoplasmic proteins were extracted using corresponding commercial kits (Thermo，78835)，Nuclear membrane proteins were extracted with a Nuclear Envelope Protein Extraction Kit (Invent Biotechnologies) according to the provided protocol. Protein samples were separated by SDS-PAGE, transferred to PVDF membranes, blocked with 5% skim milk, and incubated overnight at 4°C with primary antibodies against MPO, CD63, LaminB1, and GAPDH. Membranes were then incubated with HRP-conjugated secondary antibodies, and bands were visualised via enhanced chemiluminescence.

**ELISA for intranuclear and cytoplasmic MPO**

Concentrations of MPO in extracted nuclear and cytoplasmic fractions were measured using a commercial human MPO ELISA Kit (R&D Systems, DMYE00B) per the manufacturer’s protocol. Absorbance was detected at 450 nm with a microplate reader, and MPO concentrations were calculated against a standard curve.

**Dynamic monitoring and trajectory analysis**

This exploratory subgroup analysis included only patients with radiologically and microbiologically confirmed pulmonary infections (community-acquired or hospital-acquired pneumonia), while excluding other infection foci (e.g., intra-abdominal, bloodstream, urinary tract, or skin/soft tissue) to reduce heterogeneity in neutrophil activation. This analysis was restricted to pulmonary infections for two main reasons. First, pneumonia was the most prevalent source of infection in the ICUs of both participating centers, providing the largest eligible patient pool for consistent daily blood sampling over a full 7-day follow-up period. Second, pulmonary infection typically drives a relatively sustained and stable pattern of neutrophil activation without the abrupt inflammatory fluctuations seen in postsurgical intra-abdominal infections or rapid pathogen clearance in uncomplicated bloodstream infections, allowing us to characterize complete nmCD63 trajectories with fewer confounders. Peripheral blood samples were collected daily within 7 days of enrolment to measure nuclear membrane CD63, along with conventional clinical biomarkers (CRP and PCT) and SOFA scores.

Based on the 7-day outcomes, patients in the NS-I group were stratified into improved, progression to sepsis, or stable groups. Patients in the S group were classified as stable, non-survival, or transferred to the general ward. Clinical outcomes at day 7 were operationalized as follows: "improved" was defined as a decrease in SOFA score by ≥2 points from baseline or discharge to a general ward; "stable" was defined as a SOFA score change of <2 points without new organ support; "progressive" was defined as an increase in SOFA score by ≥2 points, initiation or escalation of organ support (mechanical ventilation, vasopressors, or renal replacement therapy), or death. These criteria were applied identically to both the NS-I and S cohorts. For each group, the dynamic trajectories of nuclear membrane CD63 and the temporal relationship between CD63 changes and their temporal associations with plasma MPO–DNA complexes (as markers of NETs), the oxygenation index, and conventional biomarkers were analyzed.

**Reference Standard and Clinical Classification**

Infection status was determined by two attending ICU physicians independently, through integrated review of microbiological culture results (blood, sputum, urine, and other site-specific cultures), clinical manifestations (fever, hypothermia, leukocytosis or leukopenia, purulent secretions), and radiological findings (chest radiography, CT, or ultrasound). Discrepancies were resolved by consensus with a third senior intensivist. This composite clinical reference standard was selected over microbiological culture alone because culture-negative sepsis accounts for 30–50% of all sepsis cases and would lead to substantial misclassification if culture positivity were required. Sepsis was defined per Sepsis-3 criteria as a ≥2-point increase in the SOFA score from a documented or inferred baseline, in the context of confirmed or suspected infection. Infected patients not meeting Sepsis-3 criteria were classified as non-severe infection (NS-I). Critically ill patients without evidence of infection (negative cultures, no clinical or radiological signs of infection, and an alternative non-infectious diagnosis) were classified as non-infected (NI). The alternative diagnoses in the NI group are summarized in Table S15.

**Table S15 Alternative diagnoses in the non-infected (NI) group**

| **Primary Diagnosis** | **Derivation (n=25), n (%)** | **Validation (n=10), n (%)** | **Total (n=35), n (%)** |
| --- | --- | --- | --- |
| Postoperative state (non-infectious) | 10 (40.0) | 4 (40.0) | 14 (40.0) |
| Major trauma / burns | 4 (16.0) | 2 (20.0) | 6 (17.1) |
| Acute pancreatitis | 2 (8.0) | 1 (10.0) | 3 (8.6) |
| Acute exacerbation of COPD / asthma | 3 (12.0) | 1 (10.0) | 4 (11.4) |
| Cardiovascular event (AMI, stroke) | 3 (12.0) | 1 (10.0) | 4 (11.4) |
| Autoimmune disease flare | 1 (4.0) | 0 (0.0) | 1 (2.9) |
| Other non-infectious critical illness | 2 (8.0) | 1 (10.0) | 3 (8.6) |
| Total | 25 (100) | 10 (100) | 35 (100) |

Note: The NI group comprised critically ill patients without evidence of infection. Diagnoses were abstracted from admission records and final discharge diagnoses. Categories are not mutually exclusive; patients with multiple comorbidities are classified by the primary reason for ICU admission.

### Treatment Characteristics

Data on antimicrobial therapy, fluid resuscitation, vasopressor support, mechanical ventilation, and renal replacement therapy within the first 24 hours of admission were retrospectively abstracted from electronic medical records. Treatment characteristics across the NI, NS-I, and S groups are summarized in Table S16.

### Table S16 Treatment characteristics of critically ill patients within 24 h of admission

| **Variable** | **NI (n=35)** | **NS-I (n=44)** | **S (n=57)** | **P** |
| --- | --- | --- | --- | --- |
| Antibiotics prior to sampling, n (%) | 13 (37.1) | 38 (86.4) | 56 (98.2) | <0.001 |
| Time antibiotic→sampling (h), median (IQR) | 4 (2–8) | 3 (1–6) | 2 (1–5) | 0.002 |
| Fluid resuscitation (>30 mL/kg), n (%) | 11 (31.4) | 21 (47.7) | 37 (64.9) | 0.008 |
| Vasopressor support, n (%) | 12 (34.3) | 23 (52.3) | 42 (73.7) | <0.001 |
| Mechanical ventilation, n (%) | 16 (45.7) | 27 (61.4) | 45 (78.9) | 0.003 |
| Renal replacement therapy, n (%) | 4 (11.4) | 8 (18.2) | 17 (29.8) | 0.045 |

Note: Treatment data were abstracted from electronic medical records for the first 24 hours following ICU admission. Between-group comparisons: chi-square or Fisher exact test (categorical), Kruskal-Wallis test (continuous). NI, non-infected; NS-I, non-severe infection; S, sepsis.
